# Green nanotechnology of MGF-AuNPs for immunomodulatory intervention in prostate cancer therapy

**DOI:** 10.1038/s41598-021-96224-8

**Published:** 2021-08-18

**Authors:** Menka Khoobchandani, Aslam Khan, Kavita K. Katti, Velaphi C. Thipe, Amal Y. Al-Yasiri, Darsha K. D. MohanDoss, Michael B. Nicholl, Ademar B. Lugão, Chetan P. Hans, Kattesh V. Katti

**Affiliations:** 1 Department of Radiology, Institute of Green Nanotechnology, University of Missouri, Columbia, MO 65212 USA; 2 Department of Radiation Oncology, Washington University School of Medicine, 4511 Forest Park Ave, St. Louis, MO 63108 USA; 3 Department of Biochemistry, University of Missouri, Columbia, MO 65212 USA; 4 Laboratório de Ecotoxicologia, Centro de Química e Meio Ambiente, Instituto de Pesquisas Energéticas e Nucleares (IPEN), Comissão Nacional de Energia Nuclear, IPEN/CNEN-SP, Butantã, São Paulo, SP Brasil; 5 Nuclear Science and Engineering Institute (NSEI), University of Missouri, Columbia, MO 65211 USA; 6 College of Dentistry, University of Baghdad, Baghdad, Iraq; 7 Dhanvantari Nano Ayushadi Pvt Ltd, No. 8/34, Neelakanta Mehta Street, T. Nagar, Chennai, 600017 India; 8 South Texas VA Health Care San Antonio, San Antonio, TX USA; 9 Department of Medicine-Cardiology, University of Missouri, Columbia, MO 65212 USA; 10 Department of Physics, University of Missouri, Columbia, MO 65212 USA; 11 University of Missouri Research Reactor (MURR), University of Missouri, Columbia, MO 65212 USA

**Keywords:** Cancer, Medical research, Oncology, Materials science, Nanoscience and technology

## Abstract

Men with castration-resistant prostate cancer (CRPC) face poor prognosis and increased risk of treatment-incurred adverse effects resulting in one of the highest mortalities among patient population globally. Immune cells act as double-edged sword depending on the tumor microenvironment, which leads to increased infiltration of pro-tumor (M2) macrophages. Development of new immunomodulatory therapeutic agents capable of targeting the tumor microenvironment, and hence orchestrating the transformation of pro-tumor M2 macrophages to anti-tumor M1, would substantially improve treatment outcomes of CRPC patients. We report, herein, Mangiferin functionalized gold nanoparticulate agent (MGF-AuNPs) and its immunomodulatory characteristics in treating prostate cancer. We provide evidence of immunomodulatory intervention of MGF-AuNPs in prostate cancers through observations of enhanced levels of anti-tumor cytokines (IL-12 and TNF-α) with concomitant reductions in the levels of pro-tumor cytokines (IL-10 and IL-6). In the MGF-AuNPs treated groups, IL-12 was elevated to ten-fold while TNF-α was elevated to about 50-fold, while IL-10 and IL-6 were reduced by two-fold. Ability of MGF-AuNPs to target splenic macrophages is invoked via targeting of NF-kB signaling pathway. Finally, therapeutic efficacy of MGF-AuNPs, in treating prostate cancer in vivo in tumor bearing mice, is described taking into consideration various immunomodulatory interventions triggered by this green nanotechnology-based nanomedicine agent.

## Introduction

The latest epidemiological investigation (spanning January 1, 2008–March 31, 2018) of patients with castration-resistant prostate cancer (mCRPC) has concluded high mortality suggesting a significant unmet clinical need in prolonging life span of human population inflicted with this deadly disease globally^1–4^. There is an emerging consensus that current therapies are poorly effective for patients with castration-resistant prostate cancer (CRPC), where the disease manifests from asymptomatic or minimally symptomatic, non-metastatic disease to symptomatic or highly metastatic condition, depending on the time of diagnosis with significant interpatient variation^5–7^. The United States Food and Drug Administration (FDA) has approved several chemotherapeutic agents including docetaxel, cabazitaxel, abiraterone, and enzalutamide for treating such patients. Drug resistance, attributable to modulation of myeloid-derived suppressor cells (MDSCs), is seen in a significant proportion of CRPC patients. Myeloid-derived suppressor cells (MDSCs) induce an immune suppressive microenvironment and promote the M2-polarized tumor associated macrophages (TAMs). These macrophages present remarkable ability to suppress T-cell responses thus supporting angiogenesis and metastasis of CRPC. Macrophages, the myeloid derived immune cells of the innate immune system, manifest two states of polarization (M1 and M2) that develop in direct response to different stimuli. The polarization and differentiation of macrophages into the cancer-inhibiting M1 and cancer-promoting M2 phenotypes represent the two states of macrophages in the tumor microenvironment^8–10^.

Numerous studies have also shown that tissue and serum exosomes from prostate cancer patients induced higher levels of macrophage polarization into an alternatively activated M2 (pro-tumor) phenotype^11–13^. The interaction of polarized macrophages with cancer cells plays a crucial role in a variety of cancers including prostate cancers^9,14,15^. Several investigations have provided important insights on the role of the polarization of macrophages from M1 into M2 phenotypes and how this macrophage axis is directly involved in prostate cancer initiation, progression, and metastasis^16–18^. An additional contributing factor for enhanced levels of pro-tumor M2 phenotypes in a vast majority of prostate and most solid tumors is attributed to elevated NF-κB signaling, upregulated by the release of cytokines by M2 macrophages, in the tumor microenvironment^19,20^. Compelling evidence shows that chemotherapeutic and radiation treatments of solid cancers in general, and prostate tumors in particular, activates NF-κB, a key transcription factor that plays a critical role in the development and progression of cancers and consequently aiding chemo and multi therapy drug resistance. Upregulated NF-κB activity can activate pro-survival pathways, including BCL-2. Therefore, cancer treatment emphasizing personalized therapy through immune boosting precision medicine, capable of targeting M2 macrophages, is distinguished from a plethora of “common denominator” treatment approaches in current use^21–23^.

In the context of developing novel therapies for treating drug-resistant cancers such as castration-resistant prostate cancer (CRPC), high antioxidant capacity and immunomodulatory phytochemicals are gaining considerable scientific and clinical interests^24,25^. Anti-neoplastic activity of phytochemicals mainly depends on their multi-target mechanism of action, including their ability to modulate the host immune response to cancer, reducing inflammatory microenvironment and enhancing lymphocyte oncosurveillance^26–28^. Numerous therapeutic effects of various phytochemicals are believed to be based on mechanisms of modulation of innate immunity more specifically macrophage function^29,30^. Since carcinogenesis is multi-factorial activity involving several signaling pathways, multi targeted phytochemicals therefore represent a promising therapeutic domain in oncology^31,32^. However, one of the major challenges, which continue to impede the application of phytochemicals, in cancer treatment is associated with achieving adequate bioavailability at tolerable doses. This is a vexing problem in translating promising findings from cell culture and animal models into clinically efficacious phytochemical-based drugs.

Nanotechnology offers practical and scientifically most effective means to create multitudes of signatures of phytochemicals on individual nanoparticles—thus enhancing bioavailability to achieve optimum therapeutic payloads at the tumor site^33–35^. Over the last two decades, we have successfully demonstrated that large surface area of gold nanoparticles can be embedded with a plethora of phytochemicals to create biocompatible cancer therapeutic nanomedicine agents^33–51^. Our extensive results, using tumor bearing rodents as well as in tumor bearing dogs (where the disease mimics spontaneously occurring tumors in human patients), have demonstrated optimum therapeutic efficacy using tolerable doses^33–51^. Our investigations, therefore, provide compelling rationale to develop phytochemical-embedded immunomodulatory nanomedicine agents for use in a wide array of applications in oncology^33–51^.

In view of the extraordinary importance of immunomodulatory intervention in treating mCRPC, we focused our attention on the creation of Mangiferin encapsulated gold nanoparticles (MGF-AuNPs). Mangiferin, used extensively in ancient medicine, is a glucose-functionalized xanthonoid found in large abundance in mangoes fruit peel^52^. Several studies have shown that Mangiferin exerts antioxidant activities, inhibitory effects on type II 5α-reductase in vitro, gastroprotective and also antidiabetic effects in rodents^53–57^. Administration of Mangiferin in swiss mice have shown in vivo growth-inhibitory activity against ascitic fibrosarcoma^58^. This phytochemical has demonstrated enhanced tumor cell cytotoxicity of the splenic cells and peritoneal macrophages of normal and tumor-bearing mice^59^. Mechanistic investigations have revealed that Mangiferin causes decreased matrix metalloproteinase (MMP)-7 and -9 activities with concomitant reversal of epithelial-mesenchymal transition (EMT)^60^. There is conclusive evidence that the mechanism of modulation of MMP-7 and -9, and EMT is due to the innate ability of Mangiferin to inhibit β-catenin pathway^61,62^. Enzymatic degradation, in vivo*,* has impeded the clinical applications of this important immunomodulatory phytochemical in oncology.

Our hypothesis was that encapsulation of Mangiferin (MGF) on gold nanoparticles would create a new nanomedicine agent, MGF-AuNP, enabling improved cellular uptake of MGF-AuNPs for exerting effective immunomodulatory intervention via targeting the tumor microenvironment. In the present article, we give a conceptual overview on how a new generation of immunotherapeutic agent derived through green nanotechnology, integrating Mangiferin phytochemical onto well-defined gold nanoparticles (MGF-AuNPs), can be developed for use in prostate cancer therapy. Interestingly, combination of gold metal with phytochemicals has been used for over 5000 years in the Indian holistic Ayurvedic Medicine^63–71^. Our green nanotechnology approach of encapsulating Mangiferin onto gold nanoparticles represents an integrative momentum to merge the best of two worlds of modern nanomedicine with the traditional Ayurvedic medicine.

We describe, herein, experimental evidence that Mangiferin functionalized gold nanoparticulate nanomedicine agent, (MGF-AuNPs), successfully manipulates the M1 and M2 polarization axis through two main approaches for applications in prostate cancer therapy: (1) specific interference with M2-like tumor associated macrophages (TAM) survival or inhibiting their signaling cascades and (2) repolarization of tumor-promoting M2-like TAMs to a tumoricidal M1-like phenotype. We also describe evidence of immunomodulatory intervention of MGF-AuNPs in prostate cancers through observations of enhanced levels of anti-tumor cytokines, such as IL-12 and TNF-α, with concomitant reductions in the levels of pro-tumor cytokines, such as IL-10 and IL-6. Additionally, we provide concrete details on cellular interrogation to establish that MGF-AuNPs target laminin receptors, over expressed on prostate tumor cells, thus presenting a compelling case for applications of this nanomedicine agent in the treatment of laminin receptor-positive human tumors for both diagnosis and therapy. Ability of MGF-AuNPs to target splenic macrophages is invoked via targeting of NF-κB signaling pathway; and thus, resulting in reeducation/polarization of macrophages from pro-tumor M2 to anti-tumor M1 macrophages. Finally, therapeutic efficacy of MGF-AuNPs, in treating prostate cancer in vivo in tumor bearing mice, is described taking into consideration various immunomodulatory interventions triggered by this green nanotechnology-based nanomedicine agent. Full mechanistic details of immunotherapeutic effects of MGF-AuNPs and how tumor microenvironment targeting ability of this nanomedicine agent will play a crucial role in prostate tumor therapy are described.

## Results and discussion

### Green nanotechnological architecture of MGF-AuNPs

**Scheme 1:**
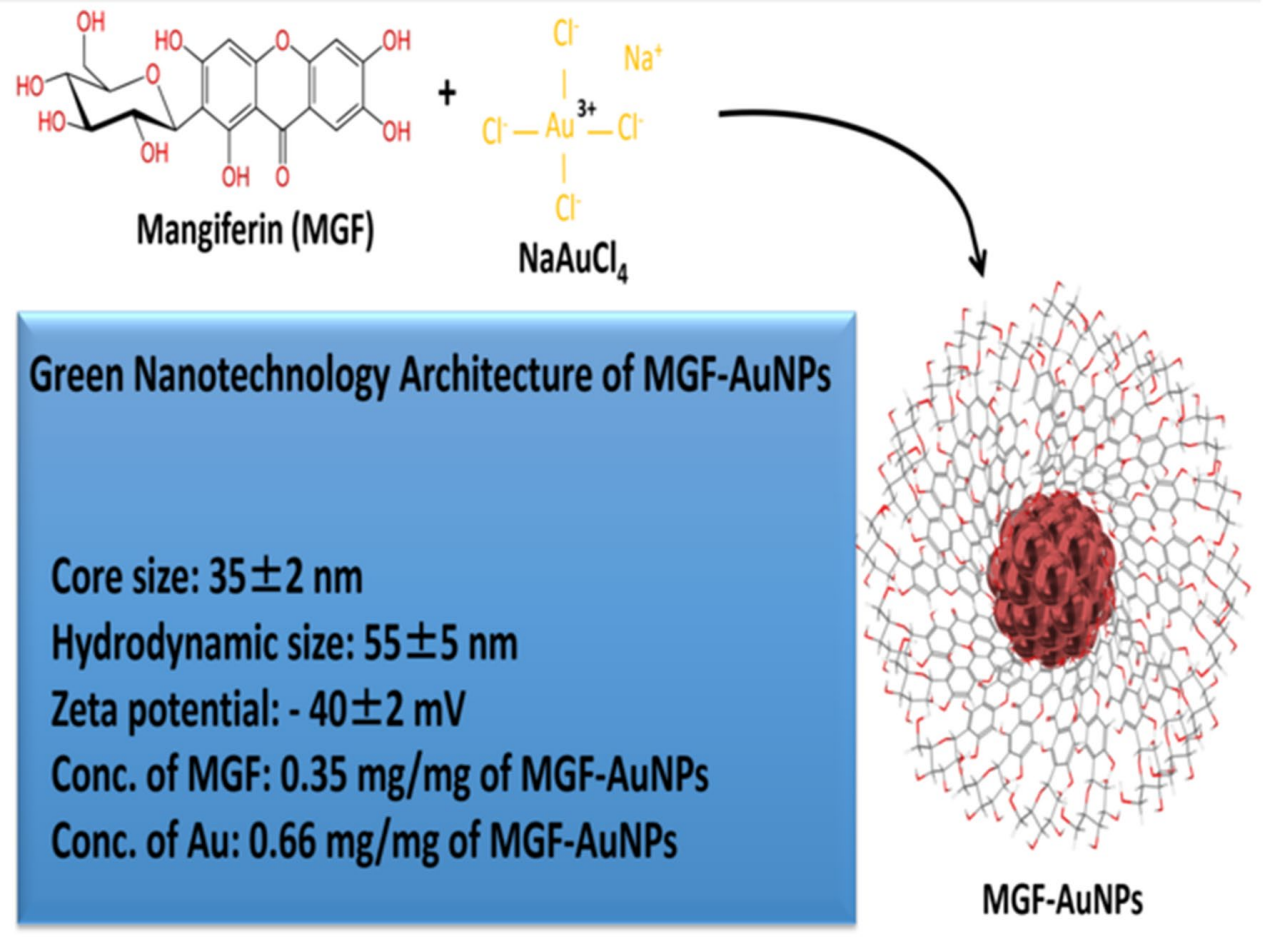
Green Nanotechnology Architecture of Mangiferin functionalized Gold Nanoparticles (MGF-AuNPs).

Mangiferin (1,3,6,7-tetrahydroxyxanthone-C2-D glucoside) is a polyphenol comprising of D-glucoside functionalized with a xanthone (Scheme 1)^72^. This phytochemical is found in abundance in the Anacardiaceae and Gentianaceae family of plant species especially in mango skin and honeybush tea^73^. Following our pioneering efforts of using Phytochemicals of plants to produce tumor specific gold nanoparticles^33–51^, we have utilized a highly innovative green nanotechnology process to functionalize Mangiferin onto gold nanoparticles to produce Mangiferin encapsulated gold nanoparticles: MGF-AuNPs (Scheme 1). Antioxidant phytochemicals can act as electron reservoirs to transform metals into their corresponding nanoparticles. The high antioxidant capacity of Mangiferin, as reflected through its oxidation potential (Epa = 0.32 V), offered a unique opportunity to use this phytochemical to transform gold salt into the corresponding nanoparticles (AuNPs). We have now optimized a highly reproducible and a scalable process wherein interaction of appropriate amounts of Mangiferin with gold salt produced the corresponding phytochemical-encapsulated gold nanoparticles (MGF-AuNPs) in aqueous media (Scheme 1). The excess Mangiferin from the reaction mixture creates a robust encapsulation around gold nanoparticles thus eliminating the need for external chemical agents for stabilization against agglomeration of MGF-AuNPs. The green nanotechnology process, as depicted in Scheme 1, offers a great example of a ‘zero carbon footprint’ process because, other than the gold salt, no other human-derived toxic chemicals were used in the overall production of MGF-AuNPs. Full characterization details of MGF-AuNPs using combinations of techniques including UV–visible Spectrophotometry, Dynamic Light Scattering (DLS), Transmission Electron Microscopy (TEM), Inductively Coupled Plasma Mass Spectrometry (ICP-MS), Energy-Dispersive X-ray Spectroscopy (EDS) and Powder X ray Diffraction (PXRD)—are all outlined in the supplementary materials section (Figures S1–S4).

### Prostate tumor cell specificity and receptor mediated endocytosis of MGF-AuNPs

We rationalized the choice PC-3 cells in our investigations based on ample evidence that suggests that PC-3 and DU-145 are androgen receptor deficient cells and that they do not respond to hormone therapy. One of the ways of treating patients with androgen receptor deficient conditions is through castration. However, this strategy does not improve the survival outcome significantly. Between PC-3 and DU-145, PC-3 tumors are more aggressive and metastatic. Androgen therapy resistant prostate tumors are more aggressive, metastatic, and fatal. Considering all these facts, we chose PC-3 cell line to develop xenograft tumors in severely combine immune-deficient (SCID) mice (see subsequent sections on therapeutic efficacy to address treatment inefficiency of current treatment strategies for androgen (castration)-resistant prostate cancers). We did not use androgen therapy sensitive prostate cancer cell line as we focused our efforts on CRPCs.

Previous studies from our laboratories have shown that polyphenolic structural motif of epigallocatechin gallate (EGCG) exhibit selective binding affinities (in the sub nanomolar ranges) with laminin receptors which are overexpressed in prostate and various other tumor cells^33,41^. We reasoned that the chemical structure of Mangiferin, which comprises of a glucose moiety (Scheme 1), should allow significantly efficient accumulation of MGF-AuNPs within tumor cells due to the Warburg effect^74,75^. We hypothesized that the Warburg effects, in conjunction with laminin receptor specificity of Mangiferin, are expected to provide selective and enhanced tumor accumulation of MGF-AuNPs in tumor cells. In order to establish laminin receptor specificity and high binding affinity of MGF-AuNPs toward laminin receptors, we have performed detailed mechanistic investigations on the endocytosis pathways of MGF-AuNPs in prostate tumor cells as discussed below.

**Figure 1:**
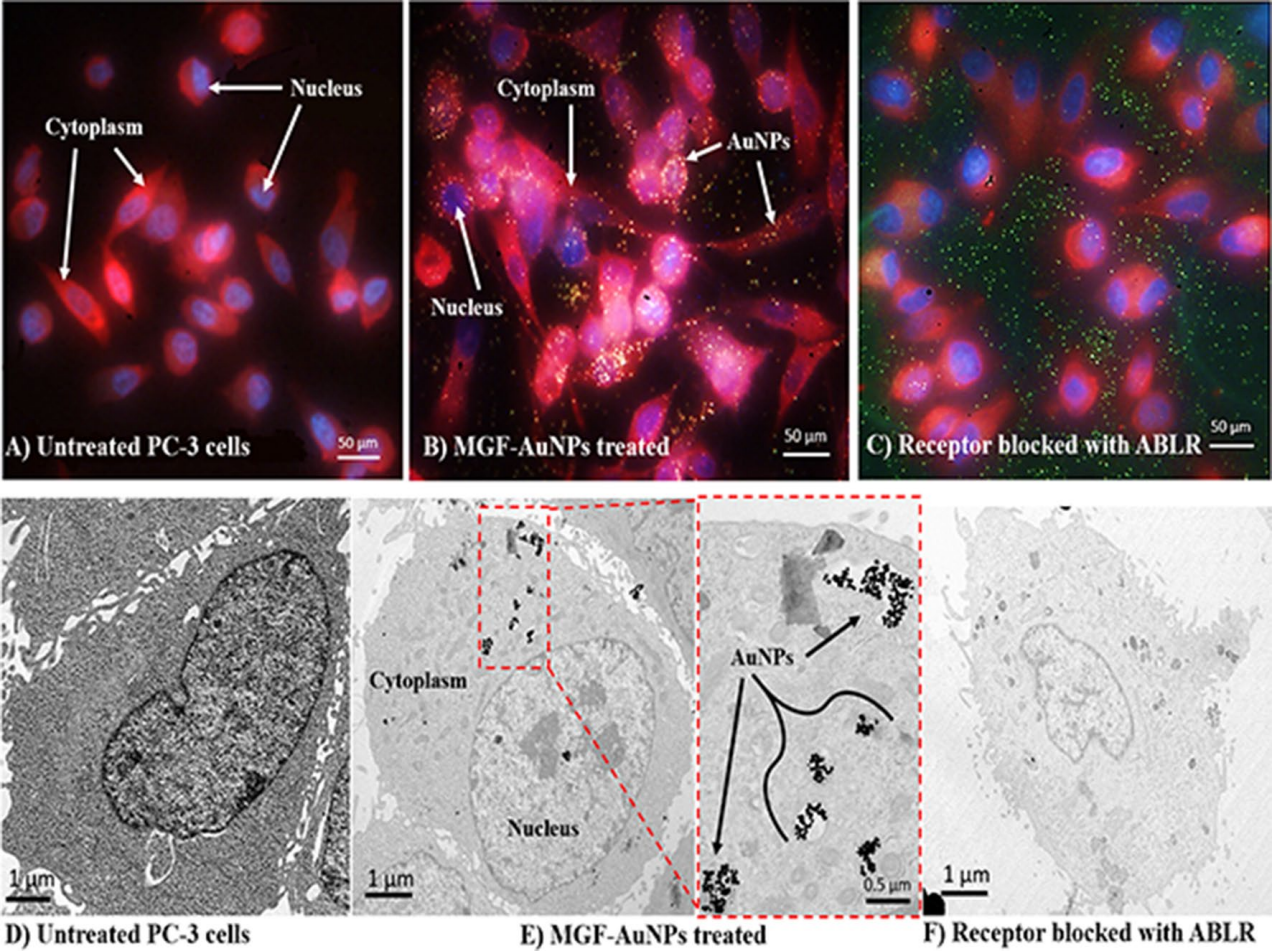
Receptor mediated endocytosis of MGF-AuNPs. (**A**,**D**): Untreated PC-3 cells; (**B**,**E**): MGF-AuNPs (41 µM) treated PC-3 cells; (**C**,**F**): Laminin receptors on PC-3 cells blocked with ABLR antibody and post treated with MGF-AuNPs, results from images **B** and **E** showing laminin receptor affinity of MGF-AuNPs in PC-3 cells. Optical images by dark field microscopy (CytoViva) and microscopic images by TEM techniques.

**Scheme 2:**
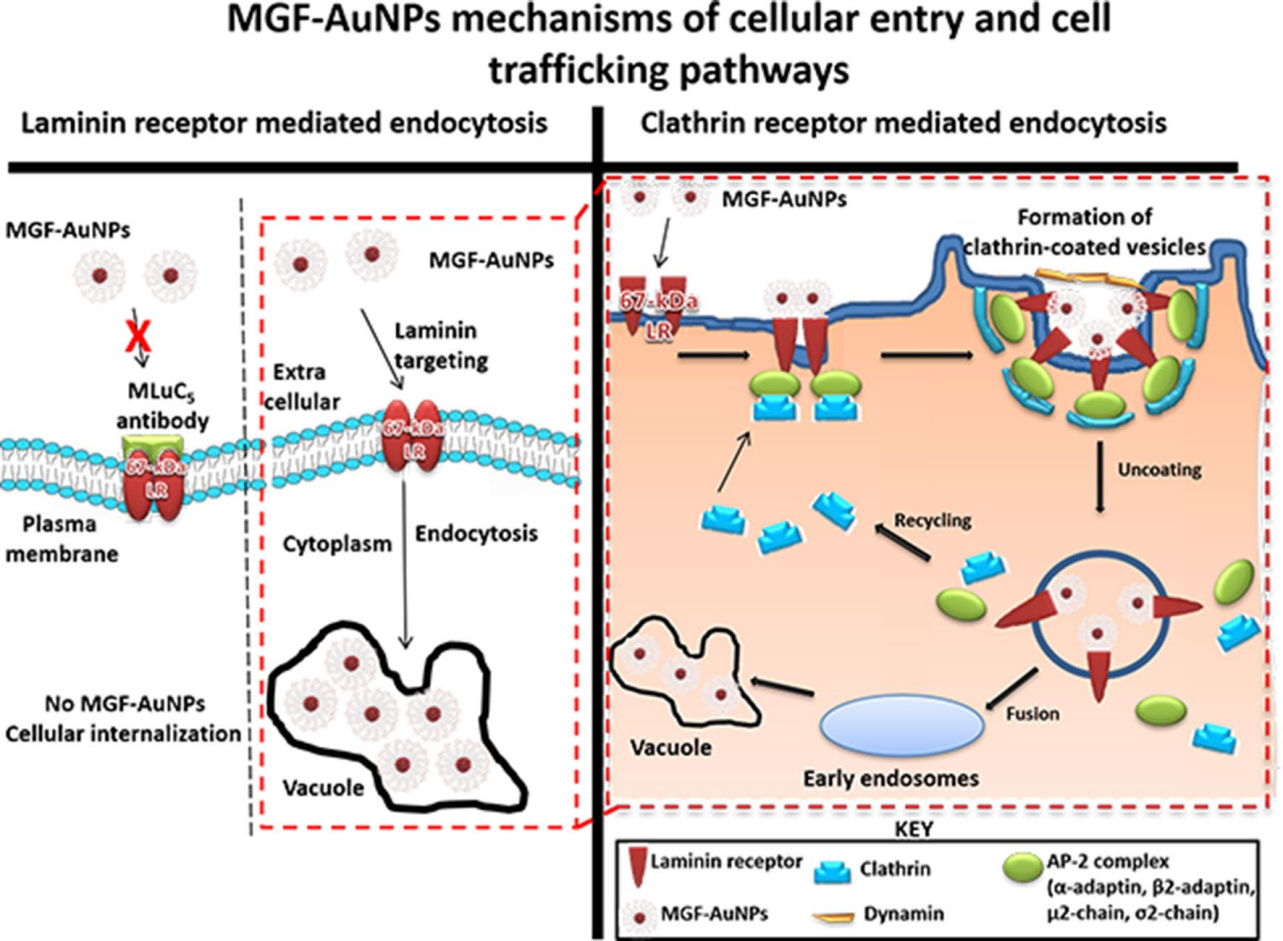
Mechanisms of endocytosis, cellular entry and cellular trafficking pathways of MGF-AuNPs into prostate tumor cells.

It is well-known that prostate tumor cells overexpress laminin receptors (67 kDa LR)^76^. Laminin receptor is an important protein involved in cell adhesion to the basement membrane as well as in the signaling transduction following this binding event^77^. In our experiments, we have probed the specificity of MGF-AuNPs toward laminin receptors that are over expressed in prostate tumor cells derived from human prostate tumors (PC-3 cells) (Fig. 1, Scheme 2). Briefly, PC-3 cells were treated with MGF-AuNPs, in two separate experiments involving the presence and absence of laminin receptor antibody (MLuC_5_), in our efforts to discern the laminin receptor mediated endocytosis of MGF-AuNPs. When the laminin receptors were not blocked by MLuC_5_ antibody, we observed very efficient endocytosis of MGF-AuNPs into PC-3 cells as shown in Fig. 1B (Dark field microscopy) and E (Transmission electron microscopy). We then saturated the laminin receptors on PC-3 cells using MLuC_5_ antibody and subsequently allowed these cells to interact with MGF-AuNPs. Microscopic analysis of these cells clearly revealed inhibition of MGF-AuNPs to internalize into PC-3 cells as shown in Fig. 1C (Dark field microscopy) and F (Transmission electron microscopy). We rationalize our findings on the basis that pre-incubation of PC-3 cells with MLuC_5_ antibody saturates Lam 67 receptors on prostate cancer cells and thus reduces or eliminates the ability of MGF-AuNPs to bind to laminin receptors on these cells. The above results, of pre- and post-blocking, of laminin receptors by MLuC_5_ antibody—taken together—unequivocally reveal that Mangiferin corona, on MGF-AuNPs, serves as a powerful laminin receptor targeting agent. These results, therefore, provide compelling evidence that MGF-AuNPs have the potential for use as tumor specific gold nanoparticles in molecular imaging and therapy of various laminin receptor-positive tumors.

**Figure 2:**
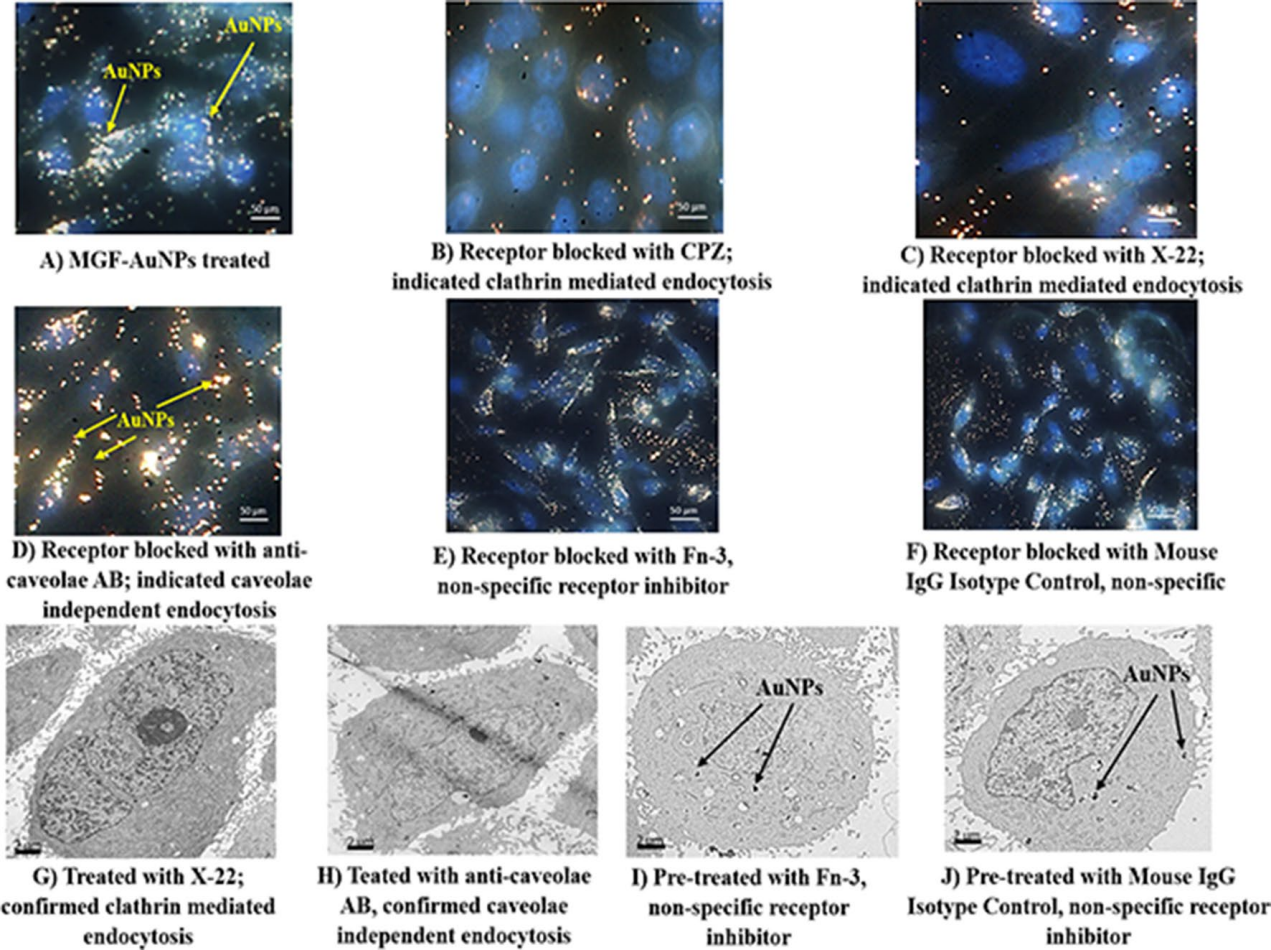
Clathrin mediated endocytosis of MGF-AuNPs (**A**–**F**): Dark field (CytoViva) microscopic images; (**G**–**J**): TEM Images showing PC-3 cells pretreated with Chlorpromazine, anticlarthrin AB, anti-caveolae AB, anti-fibronectin AB, and mouse IgG Isotype control AB, followed by treatment with MGF-AuNPs (41 µM). Images infer clathrin dependent and caveolae independent pathways for the endocytosis of MGF-AuNPs in PC-3 cells.

In order to further establish tumor cell specificity of MGF-AuNPs, we have performed laminin receptor blocking experiments using a non-specific antibody, anti-fibronectin AB (Fn-3), and mouse IgG Isotype as a control. Our selection of these antibodies in blocking experiments is based on prior evidence that they do not have known specificity towards laminin receptors on PC-3 cells. In these experiments, PC-3 cells were pre-treated with both the antibodies separately followed by treatment with MGF-AuNPs for 60 min. Post incubation, the dark field microscopic and TEM analysis of tumor cells indicated that these antibodies failed to block the endocytosis of MGF-AuNPs within PC-3 cells (Fig. 2E,F,I,J). In fact, the high propensity of endocytosis of MGF-AuNPs into PC-3 cells, post incubation with anti-fibronectin AB (Fn-3), and mouse IgG Isotype, was very similar to the results we have observed with the unblocked PC-3 cells (Fig. 2A). These detailed cellular interrogation investigations, as described above, clearly establish that MGF-AuNPs target laminin receptors on prostate tumor cells and thus reinforce their prospects for applications in the treatment of prostate and related tumors.

### Clathrin vs caveolae-mediated endocytosis and cell trafficking pathways of MGF-AuNPs

**Figure 3:**
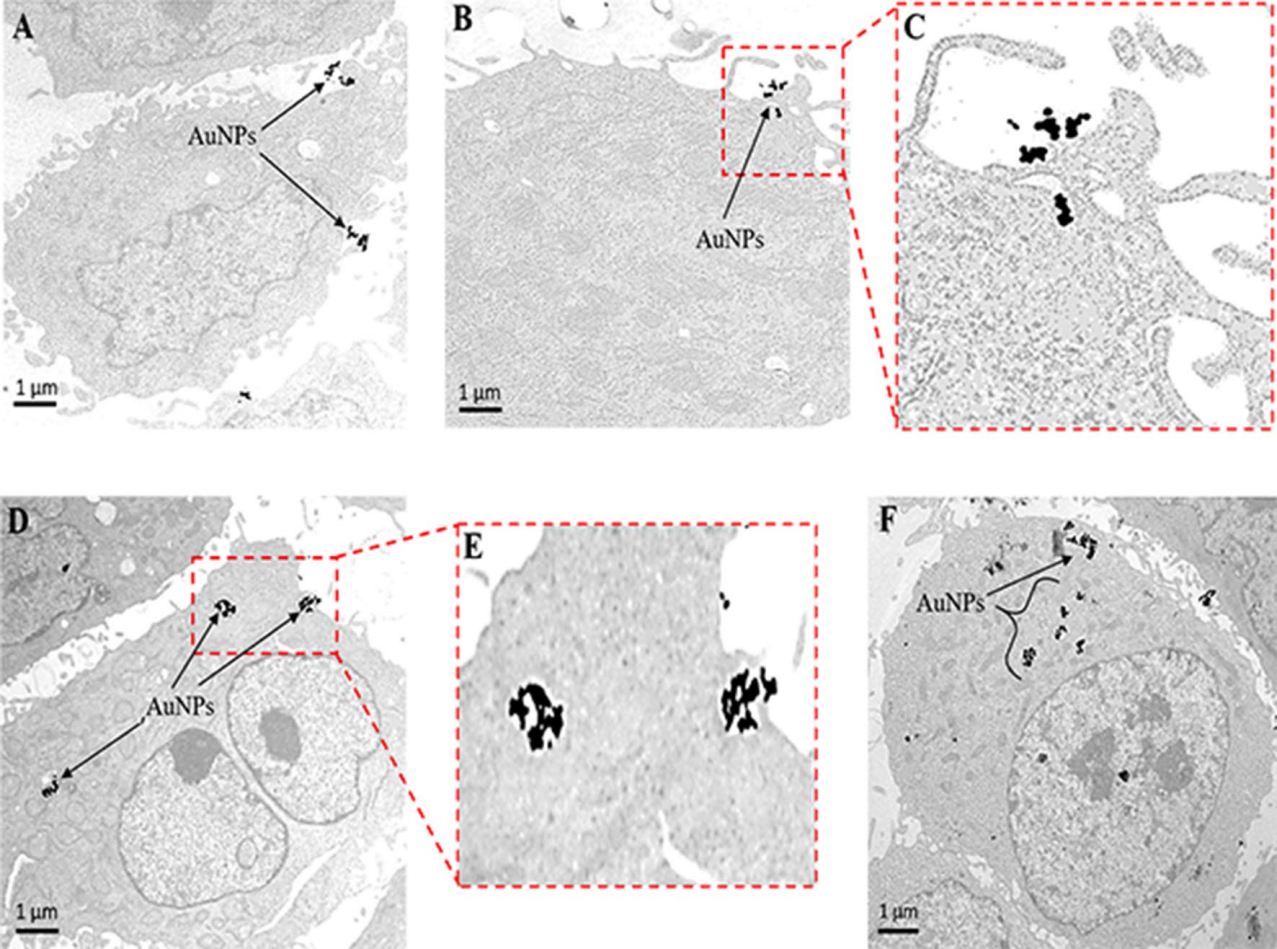
Time dependent internalization of MGF-AuNPs (41 µM) into PC-3 cells, images observed by TEM. (**A**) At 30 min; (**B**,**C**) at 60 min; (**D**,**E**) at 90 min; (**F**) at 120 min.

In order to understand the precise nature of the interaction MGF-AuNPs with prostate cellular (PC-3) membrane, we have explored further on the mode of endocytosis using two independent techniques involving dark field microscopy and transmission electron microscopy (TEM). The internalization and uptake of MGF-AuNPs with PC-3 cells were studied by incubating nanoparticles at various dilutions at select time points. Microscopic analysis of tumor cells, post incubation periods, revealed that MGF-AuNPs bind to prostate cell membrane within 30 min and internalize into the cells within 60 min of incubation time (Fig. 3A–F). Once the nanoparticles are accumulated on the cell membrane, these tumor cells appear to form a cavity like structure on the cell membrane to engulf the AuNPs (Fig. 3C). These detailed time dependent studies suggested that MGF-AuNPs internalize into the tumor cells presumably through clathrin/caveolae mediated endocytosis. Generally, clathrin and caveolae mediated endocytosis follow receptor-mediated tumor-specific pathway whereas phagocytosis or pinocytosis follow non-specific pathways. In order to confirm that MGF-AuNPs are internalized through clathrin and/or caveolae mediated tumor specific endocytosis, and not through the non-specific phagocytosis or pinocytosis pathways, we performed additional experiments involving pre-blocking clathrin coated pit using ‘chlorprompazine (CPZ)’ reagent followed by incubation with MGF-AuNPs. Microscopic examinations of tumor cells from these clathrin blocking experiments revealed that significantly lower quantitates of MGF-AuNPs are internalized into tumor cells as shown in Fig. 2B,C,G. These results clearly suggested that the mechanism of cell surface receptor uptake, and subsequent internalization of MGF-AuNPs, is mediated through clathrins. This is an important observation because clathrin mediated endocytosis are primarily responsible for subsequent intracellular downstream signaling and modulation of endocytic trafficking (discussed in subsequent sections)^78,79^. Our observations of clathrin-mediated endocytosis of MGF-AuNPs is of vital significance in prostate tumor therapy because recent investigations have shown that clathrin-mediated internalization of Cadherin-11 (Cad11) regulates surface trafficking of Cad11. It is well-known that Cadherin-11 cell adhesion molecule plays an important role in prostate cancer cell migration and that migratory function of Cad11 in prostate cancer cells is regulated through dynamic turnover of Cad11^79^.

We further focused our attention to test if MGF-AuNPs are also following caveolae-mediated pathway for internalization within prostate tumor cells. Several pre-clinical and clinical investigations have suggested that expression of Caveolin-1 (Cav-1), an integral membrane protein expressed in two isoforms (Cav-1α and Cav-1β), as a significant prognostic marker for prostate cancer^80^. Cav-1 is overexpressed in prostate cancer cells and is associated with the progression, cell survival and angiogenic activities of the disease^81^. Therefore, we reasoned to explore if the efficient endocytosis of MGF-AuNPs, as depicted in Fig. 2D,H, is mediated through caveolae pathway. Towards this objective, we performed experiments to first block caveolae on prostate tumor cells by incubating them with Anti-Caveolin-1 antibody. Post blocking of caveolae on prostate tumor cells, we incubated these cells with MGF-AuNPs and subsequently performed detailed microscopic analysis. Dark field microscopic images of PC-3 cells with and without caveolae blocking, as shown in Fig. 2D,H, suggested little/no difference, between the pre and post caveolae blocking, in the amounts of nanoparticles that were internalized. These findings, therefore, revealed that the mechanism of endocytosis of MGF-AuNPs in PC-3 cells is not mediated through caveolae expression and indeed occurs primarily through clathrin mediation as described above.

Tumor targeting capabilities of MGF-AuNPs, as shown through extensive prostate tumor cell trafficking assays outlined above, prompted us to test the potential toxicity of these nanoparticles toward normal cells. The results are summarized in the following sections.

### Interaction of MGF-AuNPs with normal cells

**Figure 4:**
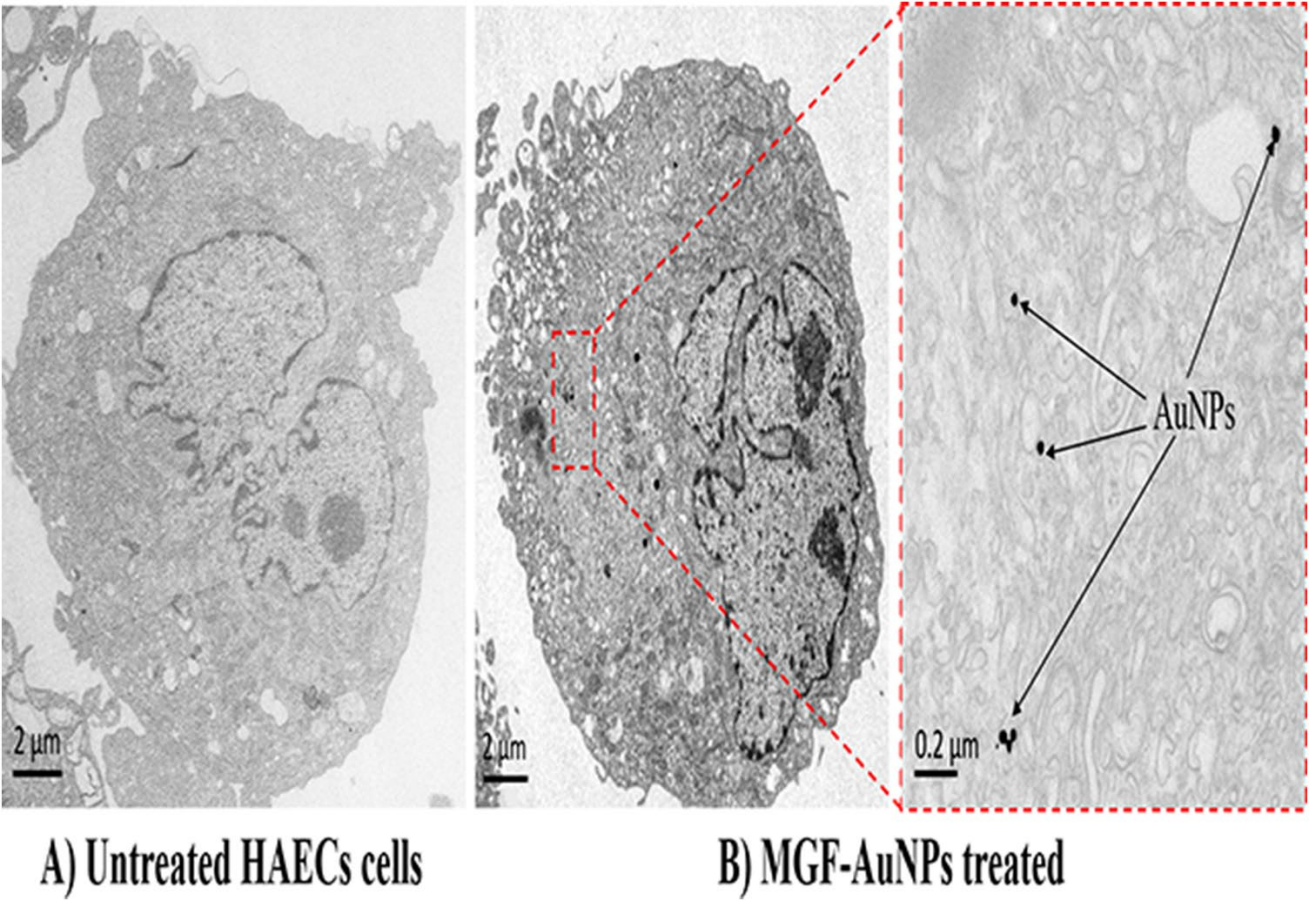
TEM images showing minimal uptake of MGF-AuNPs into human aortic endothelial cells (HAECs), 60 min post treatment of MGF-AuNPs (41 µM). (**A**) HAECs cells control; no treatment; (**B**) MGF-AuNPs treated cells (41 µM).

In order to elucidate that MGF-AuNPs are tumor cell specific and that they have minimal or no affinity toward normal cells, we have further evaluated cellular interaction of MGF-AuNPs using human aortic endothelial cells (HAECs). We hypothesized that MGF-AuNPs selectively target prostate tumor cells due to their overexpression of laminin receptors and that they cause minimal/no toxicity to normal cells because normal cells exhibit minimum laminin receptor density^82^. Therefore, we incubated MGF-AuNPs with endothelial cells (HAECs) and looked for the uptake of gold nanoparticles in these cells through electron microscopy. The results presented in Fig. 4A,B, confirmed that HAECs showed minimum uptake of MGF-AuNPs at the same dose and time point (41 µM; 60 min incubation) as was used for similar experiments with prostate tumor cells (PC-3). These results are of profound importance in the context of various applications of MGF-AuNPs as a tumor specific therapeutic agent with minimal/no toxicity to normal cells.

These detailed cellular interrogation investigations, as described above, have clearly established that MGF-AuNPs target laminin receptors on prostate tumor cells and that they exhibit minimal/no toxicity to normal cells. Exploring the effects of MGF-AuNPs on prostate tumor and normal HAECs cell viability was the next logical step in our quest to validate the applicability of MGF-AuNPs as a tumor specific nanomedicine agent.

### Effects of MGF-AuNPs on prostate tumor and normal HAECs cell viability

**Figure 5:**
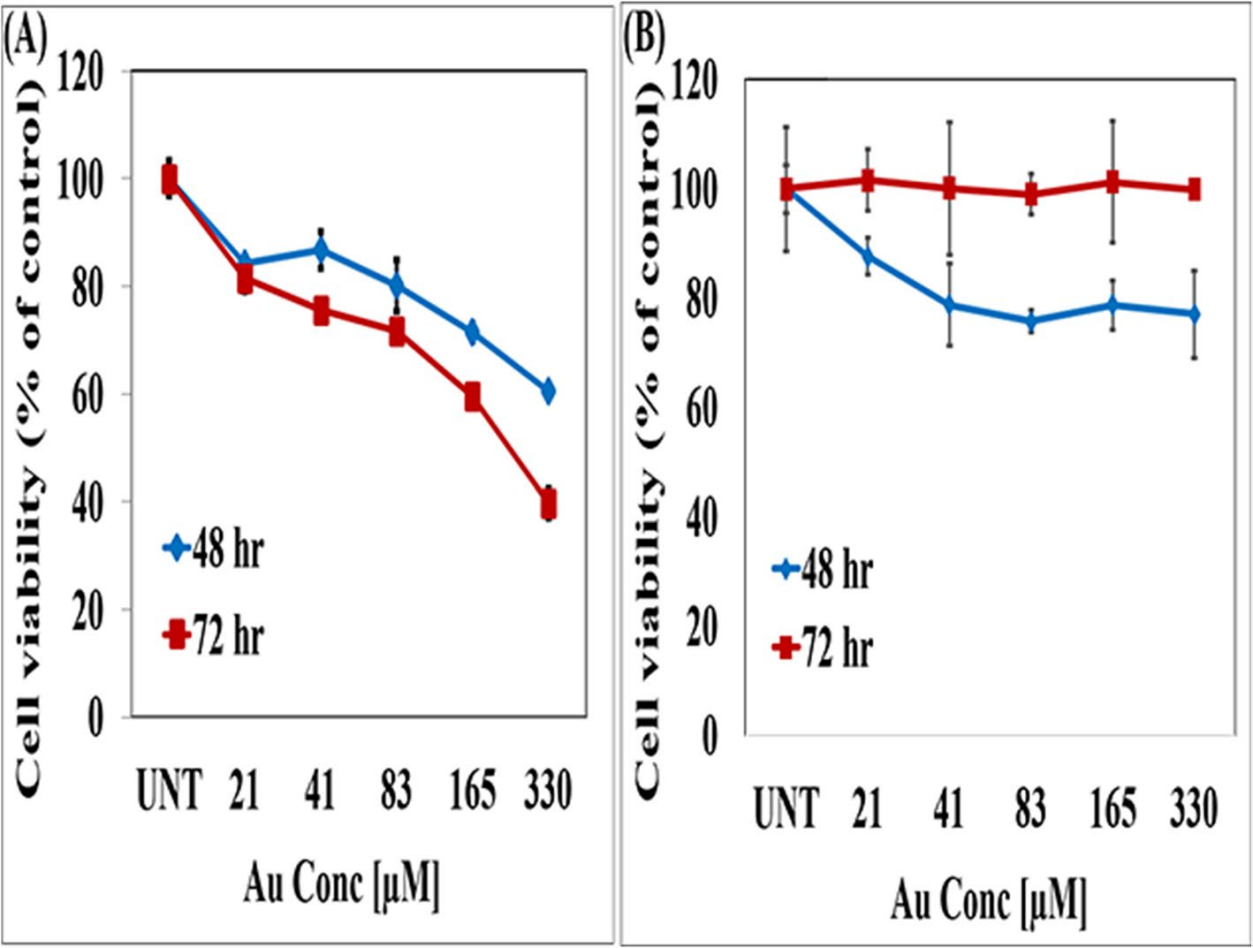
MGF-AuNPs inhibit the proliferation of PC-3 cells and not HAECs. (**A**) PC-3 cells were cultured in 96 well plates overnight for adherence and rest. The cells were treated with indicated doses of MGF-AuNPs and for 48 and 72 h. MTT assay was performed at the end of the treatment. (**B**) HAECs were cultured overnight in 96 well plates for rest and adherence. The cells were treated MGF-AuNPs for indicated times and doses. MTT assay was performed at the end of the treatment.

We have performed MTT assays to evaluate viability of prostate tumor and HAECs cells upon treatment with MGF-AuNPs. Choice of PC-3 cells was rationalized based on their innate metastatic nature. Serial dilutions of MGF-AuNPs were prepared in RPMI media to treat with PC-3 cells. The cell viability profiles, as shown in Fig. 5A, inferred that these nanoparticles exhibited dose dependent efficacy for causing death of cancer cells. Figure 5A depicts increased reduction in cancer cell viability with increasing concentrations of the MGF-AuNPs agent over a period of 48 and 72 h. At each of the concentrations, we observed reduced cell viability as compared to the control untreated, and a significant reduction of tumor cells was noted at a concentration of 165 µM and beyond. Starch stabilized gold nanoparticles (S-AuNPs) as well as Gum-Arabic stabilized gold nanoparticles (GA-AuNPs) were used as control NPs group for the in vitro experiments in all the cell viability assays to demonstrate minimal/no effect of control group of nanoparticles on cells (For details, see supplementary materials section; Figure S5).

In summary, MTT cell viability assay for prostate tumor cells and normal cells (Fig. 5A,B), taken together, with results from control group of gold nanoparticles (Figure See supplementary materials section; Figure S5), demonstrated that MGF-AuNPs presented dose limiting selective toxicity to tumor cells with no effect on normal cells.

### Evaluation of induction of apoptotic vs necrotic cancer cell death patterns of MGF-AuNPs on PC-3 cells

**Figure 6:**
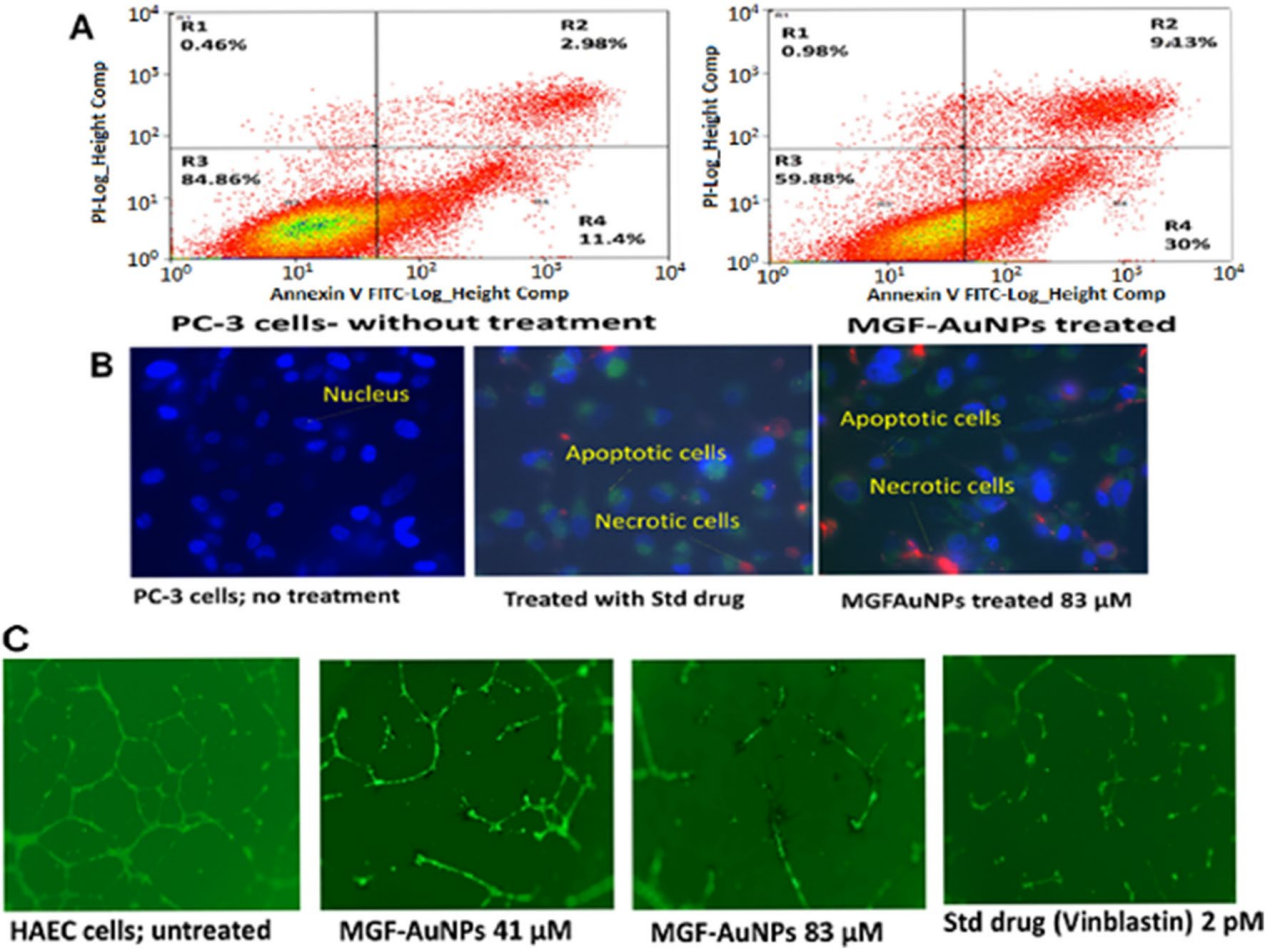
MGF-AuNPs produced apoptosis in PC-3 cells and inhibits endothelial cell tube formation: (**A**,**B**) PC-3 cells were cultured in 6 well plates for overnight followed by treatment with MGF-AuNPs for 24 h. The cells were harvested and permeabilized. After permeabilization the cells were stained with Annexin V for membrane damage and with Propidium Iodide (PI) for DNA damage. The cells were washed and analyzed by either flow cytometry or fluorescent microscopy; (**C**) 6 well plates were layered with Matrigel and HAECs were cultured on the Matrigel for tube formation. AuNPs at 41 and 83 µM for 24 h were added to the culture. Tube formation was observed under the light microscope and pictures were taken.

We wanted to test if the mechanism of tumor cell death, when MGF-AuNPs interacted with PC-3 cells, is driven through a regulated programmed cell death (apoptosis), or through a passive, uncontrolled necrosis course. Apoptotic cells are measured by their cell membrane disruption, chromatic condensation, and DNA degradation which leads to cell death. In our assays, we used flow cytometry and fluorescent microscopic techniques to visualize patterns of early and late apoptosis by PI and FITC-Annx V staining. Cells with early apoptosis are FITC (+ ve) and PI (− ve), whereas cells at late apoptotic stages are FITC (+ ve) and PI (+ ve). Our results have confirmed that PC-3 cells treated with MGF-AuNPs showed 40% total cell death including early and late-stage apoptosis at a dose of 83 µM compared to untreated controls (14.38%) (Fig. 6A) which is 278% more than the control cells. These results provide important insights that MGF-AuNPs exert apoptotic influence on PC-3 cells in tumor selective therapy. We have further verified, the Annexin V/PI assay results, through careful observations of cellular morphology of PC-3 cells upon treatment with MGF-AuNPs. The results presented in Fig. 6B indicated that cells treated with MGF-AuNPs exhibited significantly more apoptosis compared to untreated control cells. Early apoptotic cells are colored in green whereas late stage apoptotic and/or necrotic cells are colored in red (Fig. 6B). It may be discerned, from Fig. 6A,B that more cells were found in early and late apoptotic stages, in the MGF-AuNPs treatment group, as compared to the untreated control group. It is also significant to recognize that MGF-AuNPs, at the dose of 83 µM, showed almost similar pattern of apoptotic cells death as was observed upon treatment of cancer cells with the standard drug ‘Staurosporine’ (Fig. 6B). In summary, all our results, taken together, unequivocally, confirmed that MGF conjugated-AuNPs induced apoptosis of cancer cells through early-stage apoptotic phase, and finally resulting in effective programmed cancer cell death.

### Anti-angiogenesis activity of MGF-AuNPs

Angiogenesis plays a vital role in the overall growth process of cancers as it dictates the migration and differentiation of endothelial cells, which line the inside wall of blood vessels^83^. Chemical signals in the body control the rapidity of angiogenesis by providing efficient blood supply that stimulate angiogenesis as well as stimulate nearby normal cells to produce angiogenesis signaling molecules^84^. The ligand-receptor pairs such as vascular endothelial growth factor (VEGF)-VEGFR (receptor), platelet derived growth factor (PDGF)-PDGFR, fibroblast growth factor (FGF)-FGFR, and epidermal growth factor EGF-EGFR affect the angiogenesis^85^. Therefore, angiogenesis inhibiting agents are pivotal in the effective treatment of various cancers, particularly the solid tumors. We have, therefore, performed additional studies to elucidate the anti-angiogenesis capabilities of MGF-AuNPs through capillary tube structures formation assay. Phase contrast microscopic images, as depicted in Fig. 6C, clearly showed that the HAECs cells, pre-incubated with MGF-AuNPs nanomedicine agent, at 41 and 83 µM doses effectively inhibited the formation of capillaries as compared to the control untreated group, where the complete vasculature structure was seen intact (Fig. 6C). Angiogenesis plays a critical role in the growth of cancers because solid tumors need blood supply if they are to grow beyond a few millimeters in size^83^. Tumors can actually cause this blood supply to form by giving off chemical signals that stimulate angiogenesis. Tumors can also stimulate nearby normal cells to produce angiogenesis signaling molecules^84^. Vinblastine has emerged as an effective microtubule destabilizing agent because of its ability to target tubulin, thus inhibiting its polymerization and the subsequent association of microtubules. The superior antiangiogenic features of vinblastine restrain the tumor growth while decelerating malignant angiogenesis in a vast majority of human cancers^86,87^. Therefore, we have compared the antiangiogenic characteristics of MGF-AuNPs with the FDA approved vinblastine. Our results, as depicted in Fig. 6C, compellingly infer that the anti-angiogenesis effects of MGF-AuNPs are comparable with the FDA approved drug vinblastine. These findings provide compelling evidence on the vast potential of MGF-AuNPs for use as an anti-angiogenesis agent in oncology.

### Role of nuclear factor kappa B (NF-κB) transcription factor in prostate cancer

In the context of prostate cancer, several clinical investigations, involving human prostate cancer patients, have shown strong correlations between increased frequency of NF-κB p65 and a risk of disease progression^88^. Indeed, the identification of patients with high-risk prostate cancer (PC) and its direct association with the nuclear localization of NF-κB p65, from cohorts of patients, has generated considerable interest in the tremendous prognostic clinical value of this cell signaling pathway as a potential prognostic parameter in gauging treatment outcomes of advanced stage prostate cancer patients^89^. Therefore, we have probed the potential utility of MGF-AuNPs as a NF-κB targeting agent through interactions with PC-3 prostate tumor cells from the human prostate tumor origin.

### MGF-AuNPs target nuclear factor kappa B (NF-κB) transcription factor

Nuclear factor kappa B (NF-κB) constitute a family of genes acting in concert in malignant tumor invasion, migration and metastasis, of various human cancers including breast, colon, lung, oral, pancreatic, and prostate cancers^90,91^. Several investigations have inferred that (NF-κB) activation is directly responsible for the cross talk between inflammation and cancer progression^92^. The remarkable interrelationship of NF-κB activation to tumor progression—through a combination of processes including tumor cell proliferation, retarding apoptosis, accelerating angiogenesis, promoting pro-tumor macrophages—singularly and collectively underscore the importance of developing new therapeutic agents that target NF-κB both for the prevention as well as for the treatment of various human cancers^92,93^. We have, therefore, undertaken evaluations to examine if MGF-AuNPs can efficiently suppress the activation of NF-κB in tumor cells. Our investigations entailed seeding PC-3 cells into 6 well plates with subsequent treatment with MGF-AuNPs. These MGF-AuNPs-treated cells were subsequently post-treated with tumor necrosis factor alpha (TNF-α) for another 30 min at 37 °C. TNF-α, is a multifunctional pro-inflammatory cytokine that belongs to the tumor necrosis factor superfamily^94^. The control group PC-3 cells were incubated with TNF-α only to stimulate NF-κB^95,96^. Quantification of NF-κB suppressive effects of MGF-AuNPs was carried out using flow cytometry in comparison with the controls. The flow cytometry analysis, as shown in the supplementary materials in Figure S6, indicated that MGF-AuNPs effectively blocked the TNF-α-induced-NF-kB activation in the PC-3 cells, which were pretreated with the nanomedicine agent, with subsequent post treatment with TNF-α. Images depicted in Figure S6 further confirmed that the NF-κB levels were indeed significantly higher in the PC-3 control group—which were not treated with MGF-AuNPs (Figure S6). These studies highlight two important experimental findings that MGF-AuNPs can be used: (1) for the inhibition of NF-κB signaling thereby transferring signals to the nucleus to induce corresponding gene expression, to control excessive cell proliferation, reduce/eliminate apoptotic resistance—all resulting in anti-angiogenesis, inhibiting invasion, and thus to effectively control/eliminate metastasis; and (2) in the overall design of new targeted therapeutics aimed at cancer prevention and therapy.

TNF-*α* has gained a ubiquitous “yin and yang” role in cancer development and metastases. It is well-known that TNF-*α* released from macrophages activates NF-κB-mediated signaling pathway in various cancers—thus playing a major role in cancer progression and metastasis^94–97^. Tumor microenvironment is highly dynamic in cell-to-cell crosstalk between NF-κB and other signaling pathways. Such crosstalk feedback loops modulate the inflammatory response in macrophages by altering NF-κB activation^98^. Encouraged by the NF-κB targeting ability of MGF-AuNPs, we reasoned the logistics of the role of this nanomedicine agent in targeting tumor microenvironment. A strong rationale for such investigations stemmed from the fact that tumor-associated macrophages (TAMs) engineer regulation of cancer growth and metastases through alterations of tumor cell proliferation, migration, invasion, angiogenesis, and immunosuppression. Immune cells outside the tumors produce pro-inflammatory cytokines to activate tumor NF-κB pathway of tumor cells and also tumor-infiltrating cells such as macrophages and myeloid-derived suppressor cells (MDSCs)—all resulting in a tumor-permissive environment for the growth and metastasis.

In the following sections, we will describe the key findings of the innate ability of MGF-AuNPs to target macrophages and subsequently on how the macrophage affinity of this nanomedicine agent would translate into the design of a new immunomodulatory prostate cancer therapeutic agent.

### Target specificity of MGF-AuNPs toward macrophages

In our investigations, RAW264.7 cells were chosen over bone marrow derived macrophages (BMDMs), for two primary reasons: (1) RAW264.7 are developed from peritoneal macrophages and are, therefore, more diverse in macrophage population i.e., the macrophages come from all visceral tissues. Therefore, our overarching objective was to test if MGF-AuNPs can reeducate macrophages from diverse tissues so that this new nanomedicine agent can be used in treating tumors of different tissue origin and not just prostate tumors; (2) RAW264.7 cells are not subjected to cytokine stimulation, which is the case with BMDMs, that puts BMDMs slightly more on pre-activated side, which in turn might interfere with MGF-AuNPs ability as a macrophage re-education agent. The central hypothesis of our investigation was to address the ability of the nanomedicine agent, MGF-AuNPs, to re-educate macrophages to eliminate tumors. It is important to recognize that T cells are not involved in producing the anti-tumor effects in our investigations because we have used SCID mice which are deficient in T and B cells. Therefore, our in vivo investigations, as described in subsequent sections provide compelling evidence on the ability of MGF-AuNPs as a macrophage re-education immunomodulatory agent.

Macrophages are classified as non-neoplastic cells with pro-tumor or anti-tumor phenotypes depending on their anatomical location, and the physiological context. Classically activated macrophages (referred to as M1) and alternatively activated macrophages (referred to as M2) fit two extremes within the spectrum of the macrophage phenotypes^99^. Tumor-associated macrophages (TAMs) closely resemble “alternative” (M2) macrophages^100^. M1 macrophages are recognized as classically activated macrophages that can phagocytize pathogens and exert tumoricidal activity through activation of antitumor activity primarily by IL-12–dependent natural killer (NK) cell recruitment. On the other hand, proliferating tumors in humans exhibit polarized M2 phenotype that are directly involved in tumor metastasis, and ultimately contributing to drug resistance of the disease^101^. Tumor associated macrophages often express M2-like phenotype with high IL-10, high arginase-1 and low IL-12—all contributing to pro-tumorigenic activities. In the context of prostate cancer, there is considerable evidence supporting macrophage infiltrations (inflammation) which are associated with especially advanced stages of prostate cancer. In fact, castrated tumors possess more pro-tumorigenic M2 macrophage phenotype thus inducing the onset of immunosuppressive state^102–105^.

It is also important to note that drugs capable of targeting NF-κB signaling in TAMs can reprogram macrophages from the pro-tumor M2 to an anti-tumor M1 phenotype. This process within the TAMs promotes regression of advanced tumors by induction of macrophage tumoricidal activity and activation of antitumor activity through IL-12–dependent NK cell recruitment^106,107^. Given the importance of M2 to M1 macrophage reeducation and the established role of MGF-AuNPs in targeting NF-κB signaling, the logical next step was to evaluate the macrophage targeting ability of this nanomedicine agent, especially to explore its capability in transforming pro-tumor M2 to an anti-tumor M1 phenotype within TAMs.

**Figure 7:**
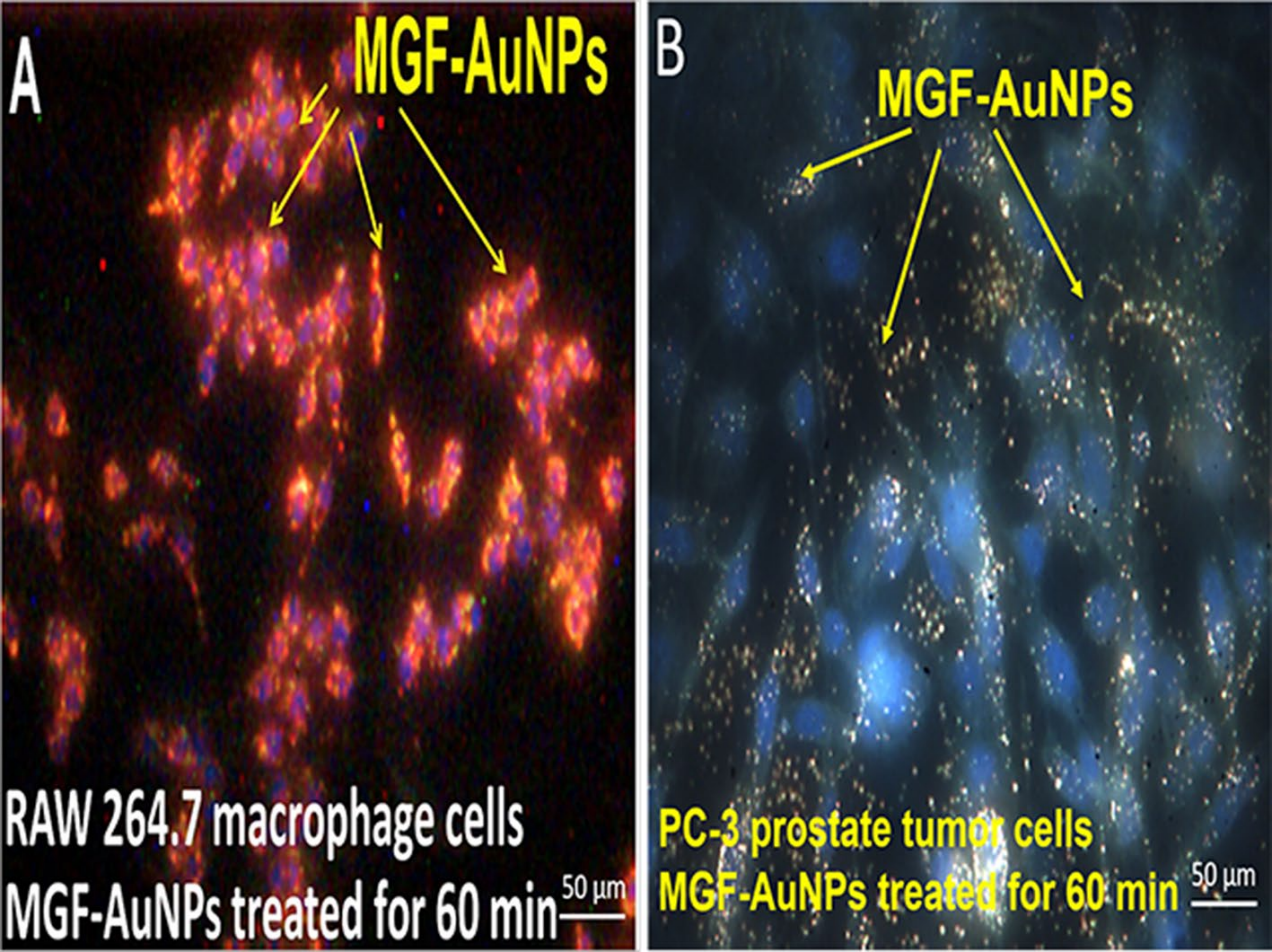
Macrophages internalize MGF-AuNPs more than PC-3 cells. (**A**,**B**) RAW 264.7 and PC-3 were cultured in 6 well plates for overnight. The cells were then incubated with MGF-AuNPs for 1 h followed by washing of the cells to remove uninternalized MGF-AuNPs. The cells were then analyzed by CytoViva dark field microscopy and pictures were taken.

In our initial experiments, we treated the RAW 264.7 macrophages with MGF-AuNPs and evaluated for the endocytosis of these nanoparticles in macrophage cells. As shown in Fig. 7, indeed, MGF-AuNPs displayed excellent affinity and propensity to internalize within macrophages through phagocytosis. It is important to note that comparison of images (Fig. 7A,B), demonstrates that, under similar experimental conditions of using MGF-AuNPs (40 µM) incubated for 60 min, macrophages assimilated higher payloads of MGF-AuNPs as compared to PC-3 cells. Macrophages phagocytized MGF-AuNPs efficiently, whereas the PC-3 cells, which use laminin receptor-mediation as the primary process to internalize these nanoparticles, exhibited significantly lower propensity for internalization of this nanomedicine agent. Having established the macrophage-avidity of MGF-AuNPs, we turned our attention in testing the ability of these nanoparticles in inhibiting NF-κB phosphorylation in macrophages.

### MGF-AuNPs inhibit NF-κB phosphorylation in macrophages

**Figure 8:**
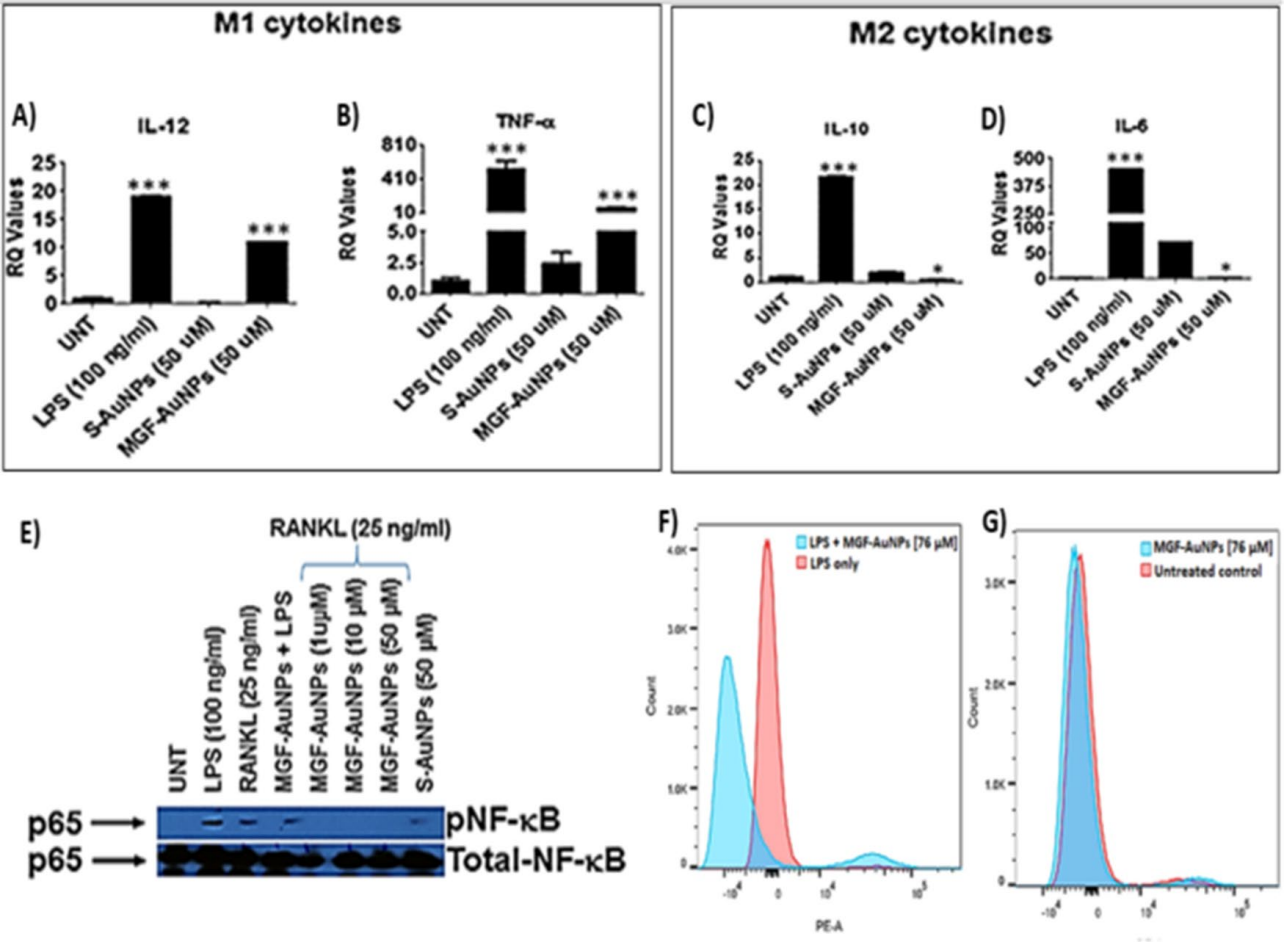
MGF-AuNPs induced polarization of macrophages and inhibits NF-κB activation. (**A**–**D**) RAW 264.7 cells were pretreated with either Starch-AuNPs (S-AuNPs as control), or MGF-AuNPs for 2 h and treated either with LPS (100 ng/mL) or RANKL (25 ng/mL) or left untreated for 4 h. RNA was isolated from treated and untreated samples and analyzed for IL-12, TNF-α, IL-10, and IL-6 by real time PCR using probes from TaqMan, Applied Biosystems. (**E**). RAW 264.7 cells were either treated with LPS (100 ng/mL) or Starch-AuNPs (S-AuNPs as control), or MGF-AuNPs or left untreated for 30 min. The cells were lysed with 1X Lamellae buffer and lysates were run on PAGE gel and transferred onto nitrocellulose membranes. The membranes were than probed for either phospho- NF-κB or NF-κB using respective antibodies. (**F**). The RAW 264.7 cells were cultured overnight in 6 well plates and pre-treated with different doses of MGF-AuNPs (0, 32 µg/mL) for 3 h. Subsequently the cells were washed and treated with LPS (100 ng/mL) for 45 min. After incubation with LPS the cells were washed, fixed and permeabilized. After permeabilization the cells were stained with PE conjugated anti-NF-κB antibody for 45 min. The cells were washed and analyzed using flow cytometry. (**G**) The RAW 264.7 cells were cultured overnight in 6 well plates and pre-treated with different doses of MGF-AuNPs (0, 32 µg/mL) for 45 min. Cells were fixed, permeabilized and stained with PE conjugated anti-NF-κB antibody for 1 h and analyzed by flow cytometry.

NF-κB is a transcription factor that resides in I**κ**B kinase (IKK) complex located in the cytoplasm along with inhibitor of NF-κB proteins (IκBs), NF-κB activation is stimulated by TNF-α, or other cell stressors which then leads to NF-κB phosphorylation and translocation to the nucleus^89^. This directly influences the transcription of pro-tumor genes in cancer cells and macrophages. The rationale for our investigations is that the NF-κB intervention in macrophages can polarize macrophages to the anti-tumor M1 phenotype to eliminate tumors^100^. In order to evaluate the ability of MGF-AuNPs to induce NF-κB inhibition in macrophages, we pretreated RAW 264.7 with either MGF-AuNPs or starch encapsulated AuNPs (Starch-AuNPs: S-AuNPs served as a control AuNPs). Our experimental findings revealed that MGF-AuNPs inhibited RANKL and LPS induced NF-κB in macrophages (Fig. 8E,F).

### MGF-AuNPs, but not S-AuNPs, polarize macrophages to anti-tumor or M1 phenotype

Tumor microenvironment modifies the macrophages which then aid in the progression of these tumors^108^. The modified macrophages or TAMs have reduced antigen presentation ability and produce elevated levels of immunosuppressive cytokines such as IL-10^108^. The macrophages within the tumor microenvironment also produce increased levels of antiangiogenic cytokines such as IL-6^109^. In order to understand the effects of MGF-AuNPs on macrophages, we have investigated the expression of macrophage polarizing cytokines such as IL-12, IL-10, IL-6, and TNF-α upon treating RAW 264.7 macrophages with MGF-AuNPs. In RAW 264.7 macrophages, which were treated with MGF-AuNPs, our experimental findings conclusively demonstrated elevated levels of anti-tumor cytokines such as IL-12 and TNF-α, while reducing the levels of pro-tumor cytokines such as IL-10 and IL-6 (Fig. 8A–D). In contrast, the results from similar investigations using the starch-stabilized gold nanoparticles (S-AuNPs), we observed no macrophage targeting capability and no influence in elevating the levels of anti-tumor cytokines such as IL-12 and TNF-α (Fig. 8). These results suggested the potential immunotherapeutic role of MGF-AuNPs and, therefore, prompted us to further probe into capabilities of this nanomedicine agent for modifying macrophages.

**Figure 9:**
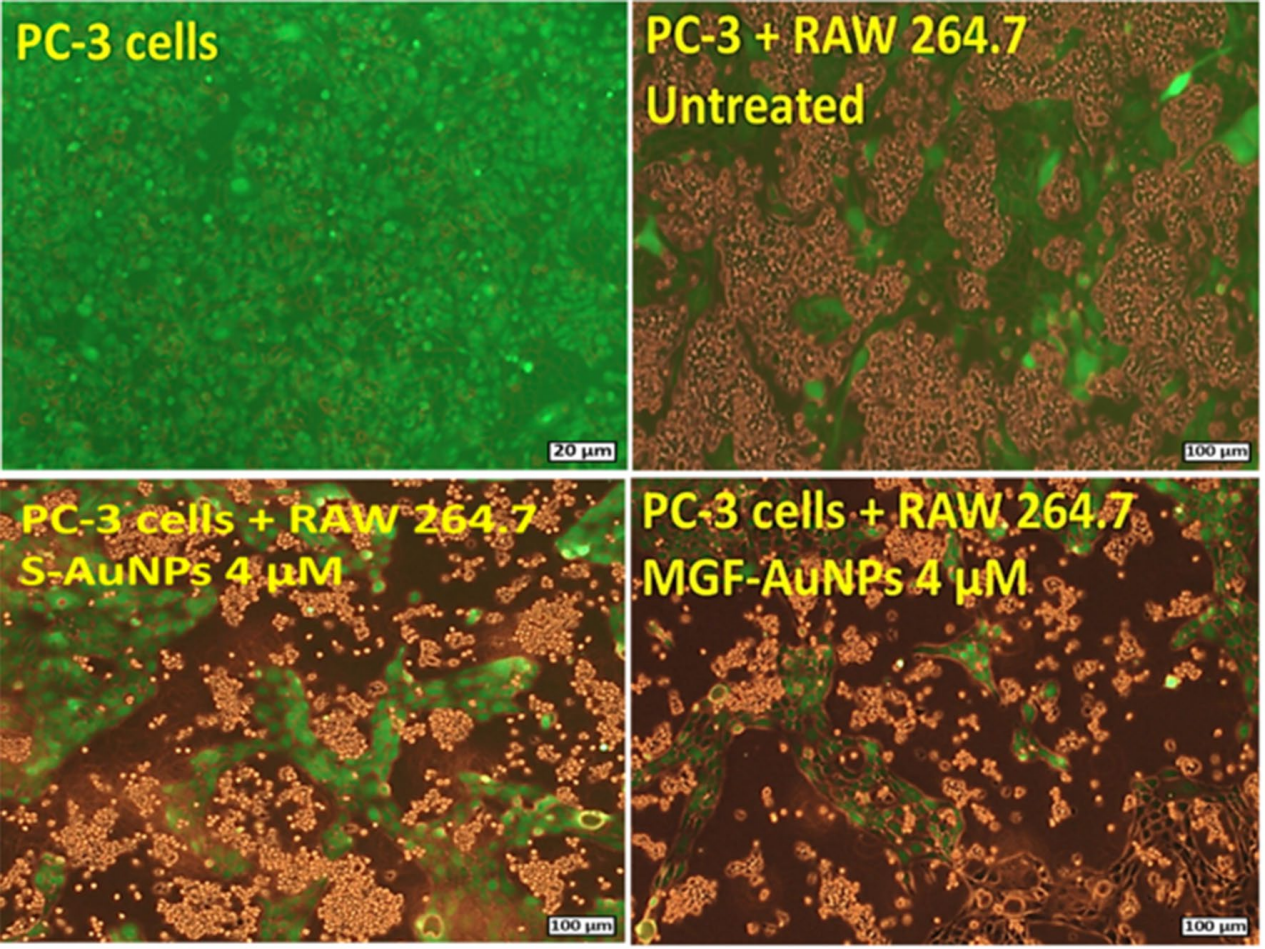
MGF-AuNPs-treated macrophages inhibited the proliferation of prostate tumor cells. Raw 264.7 macrophages were pre-treated with MGF-AuNPs for 18 h. Separately, PC-3 cells were labelled with CFSE to assess their proliferation. The macrophages were then co-cultured with PC-3 cells for 72 h. The images were obtained by fluorescent microscope.

**Scheme 3:**
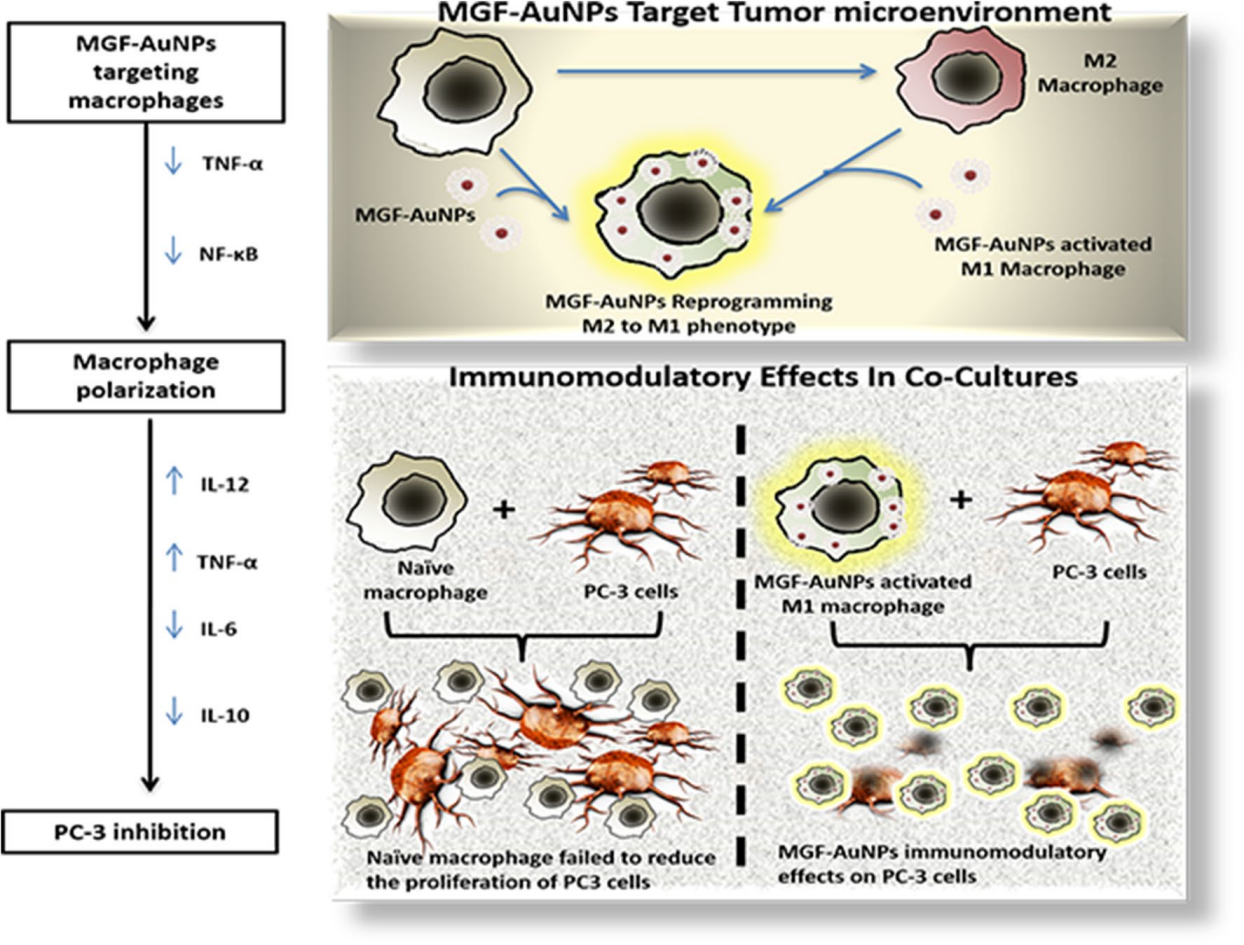
Targeting ability of MGF-AuNPs toward tumor microenvironment (TME) and it’s reprograming ability of M2 to M1 phenotype.

In order to evaluate if MGF-AuNPs can reprogram M2 macrophages into the therapeutically desirable anti-cancer M1 phenotype, we cocultured MGF-AuNPs-pretreated macrophages with prostate tumor cells (PC-3) and then looked for tumor proliferation differences between this group and the control PC-3 cells which were directly treated with naïve macrophages only (Scheme 3). We found that macrophages transfected with the MGF-AuNPs agent displayed gene expression profiles similar to anti-tumor M1 phenotype. This observation is consistent with the significant inhibition in the proliferation of tumor cells. However, PC-3 cells that were cocultured with naïve macrophages failed to reduce the proliferation of PC-3 cells (Fig. 9 and Scheme 3). These data suggest that MGF-AuNPs-mediate anti-tumor phenotype to macrophages expression and therefore warranted further investigations on whether this nanomedicine agent would promote elevation in the levels of anti-tumor cytokines such as IL-12 and TNF-α while reducing the levels of pro-tumor cytokines such as IL-10 and IL-6. With this objective in mind, we incubated MGF-AuNPs with RAW 264.7 macrophages and analyzed the levels of various pro-tumor and anti-tumor cytokines. Real-time PCR (Quantitative-PCR) showed robust increase in anti-tumor (pro-inflammatory) cytokines IL-12 (tenfold higher) and TNF-a (50-fold higher), while reducing the levels of pro-tumor cytokines such as IL-10 and IL-6 in macrophages treated with MGF-AuNPs. Similar experiments using the starch-stabilized gold nanoparticles (S-AuNPs) control showed no effects toward enhancing anti-tumor cytokines in the treated macrophage cells (Fig. 8). Tumor microenvironment modifies the macrophages which then aid in the progression of these tumors^102^. The modified macrophages or TAMs have reduced antigen presentation ability and produce elevated levels of immunosuppressive cytokines such as IL-10^108^. The macrophages within the tumor microenvironment also produce increased levels of antiangiogenic cytokines such as IL-6. Therefore, our results which demonstrate the ability of MGF-AuNPs in promoting higher levels of anti-tumor cytokines, such as IL-12 and TNF-α, while reducing the levels of pro-tumor cytokines, such as IL-10 and IL-6, are significant toward proving the immunomodulatory intervention in prostate cancer therapy (Scheme 3).

TAMs which originate from resident macrophages from the bone marrow and spleen are key tumor stromal cell types and play a critical role in tumor survival, growth, and metastasis^110,111^. Several investigations have confirmed spleen macrophages (Mφ) as the key TAM precursors, where macrophages maintain hematopoietic steady state by engulfment of neutrophils and eosinophils^112^. Tumor microenvironment modifies the macrophages which then aid in the progression of these tumors. The modified macrophages or TAMs have reduced antigen presentation ability and produce elevated levels of immunosuppressive cytokines such as IL-10. The macrophages within the tumor microenvironment also produce increased levels of antiangiogenic cytokines such as IL-6. It is important to understand how enhanced retention of MGF-AuNPs in spleen affects macrophage function.

In order to explore the ability of MGF-AuNPs to target splenic macrophages, we have undertaken a detailed biodistribution study of MGF-AuNPs in normal mice as discussed below.

### Targeting splenic macrophages

Spleen is a critical secondary lymphoid organ showing abundance of B cells, T cells, NK cells, and is also a reservoir of mononuclear phagocyte system (MPS), mainly resident macrophages^113^. Recent investigations have shown that the local and systemic immune response to cancer increases by the ability of drugs or nanoparticles to target splenic macrophages which comprise mostly of M2-like pro-tumor macrophages^114^. Macrophage position and function in splenic domains confer them unique phenotypes^115^. Suzuki et al. have shown that Gemcitabine selectively eliminates splenic Gr-1^+^/CD11b^+^ myeloid suppressor cells in tumor-bearing animals and enhances antitumor immune activity^116^. Indeed, the superior antitumoral efficacy of Trabectedin (Yondelis) has been attributed to the ability of this drug to target splenic macrophages thus exerting TAM selective cytotoxic activity towards Ly6C^high^ monocytes in circulation and in the spleen^117^. In order to evaluate further on the macrophage targeting ability and immunomodulatory characteristics of MGF-AuNPs, we have performed systemic administration via intravenous injection in mice—all aimed at testing if this nanomedicine agent is effective in targeting splenic macrophages. We discuss here compelling evidence from murine models of cancer in support of macrophage-targeted intervention strategies with the potential of MGF-AuNPs for use in dramatically reducing prostate and various other cancer morbidities through immunomodulatory mechanisms.

**Figure 10:**
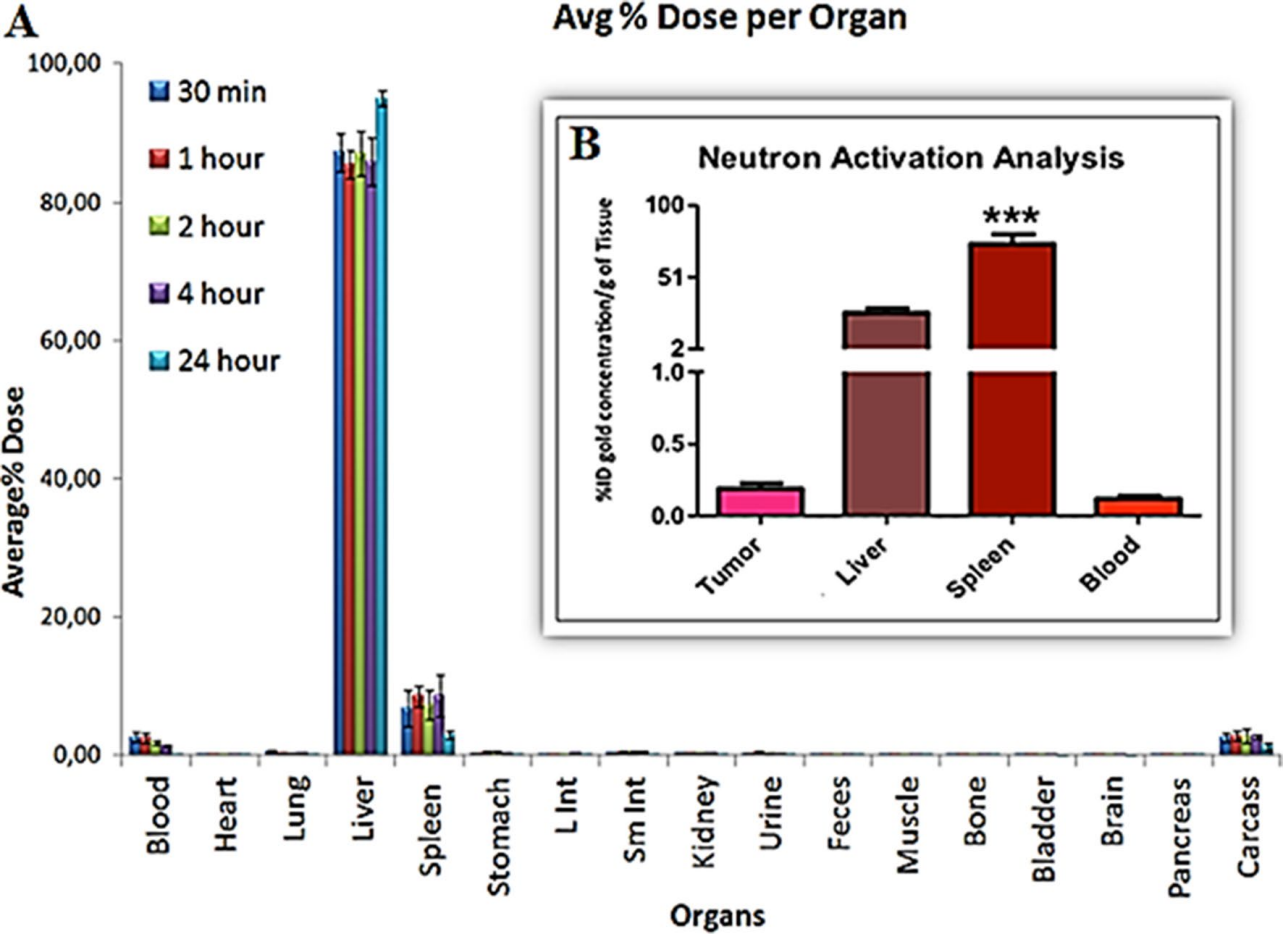
MGF-AuNPs preferentially accumulate in spleens of SCID mice: (**A**) Biodistribution of MGF-^198^AuNPs in normal mice showing selective uptake only in liver and spleen and limited/no uptake in non-target organs; (**B**) Biodistribution of MGF-AuNPs in prostate tumor bearing SCID mice. Gold concentrations in tumor, liver and spleen measured using neutron activation analysis (NAA)—showing limited uptake in tumors and major uptake in spleen and liver—suggesting targeting of MGF-AuNPs on splenic macrophages.

In order to understand the pharmacokinetics/ pharmacodynamics of MGF-AuNPs, we have evaluated the uptake of gold nanoparticles, in vivo, in normal mice as well as in prostate tumor-bearing mice. These studies were performed by first producing the radioactive equivalent of MGF-AuNPs using ^198^Au isotope because the gamma emission (0.411 MeV) of ^198^Au isotope allows scintigraphic counting of radioactivity of gold for accurate estimation of the nanomedicine agent in various organs^39^. Post administration of radioactive MGF-^198^AuNPs, in normal mice, through intravenous delivery, we analyzed for the presence of gold in various organs at various time points using scintigraphy counting (Fig. 10A)^39^. Further quantification of gold nanoparticles in spleen, liver and tumors were performed through neutron activation analysis (NAA) of various organs post administering specific amounts of MGF-AuNPs (Fig. 10B). Our results, as shown in Fig. 10A,B, showed a significant uptake of MGF-^198^AuNP in spleen. This observation is clearly in stark contrast to a vast variety of gold nanoparticles, which generally show hepatobiliary uptake in liver^34,118^. Together with these results, we infer the inherent propensity of MGF-AuNPs to target splenic macrophages.

It may be noted that the mode of delivery, going from the intravenous to intraperitoneal, did not reduce the uptake of MGF-AuNPs in splenic macrophages. The preferential accumulation in liver and spleen invoked the possibility of this nanomedicine agent to target splenic macrophages. In order to elucidate if the uptake of MGF-AuNPs in spleen is indeed of splenic macrophage origin, we have performed further in vivo investigations using severely combine immune-deficient (SCID) mice. The rationale for this animal model is based on the fact that SCID mice manifest active macrophages while lacking in T and B cells^119^. Before moving onto in vivo tumor studies with MGF-AuNPs, we tested the toxicity of this nanomedicine agent on SCID mice. The doses of MGF-AuNPs, at which prostate tumors are suppressed in vivo, caused no systemic toxicity in normal mice as elucidated through detailed toxicity studies (see supplementary materials section). Intraperitoneal administration of MGF-AuNPs in SCID mice resulted in preferential accumulation in liver and spleen which further suggested that MGF-AuNPs might be assimilated by splenic macrophages (Fig. 10). Macrophages play important role in tumor development by supporting vascularization of tumors as well as inhibition of subsequent generation of tumor specific cytokines. It is well-known that macrophages migrate to spleens to phagocytize dead RBCs, and therefore, the accumulation of MGF-AuNPs in spleen, as observed, suggests the selective accumulation of this nanomedicine agent in the macrophages.

The in vivo distribution of MGF-AuNPs in SCID and normal mice, which revealed high percentage of accumulation of MGF-AuNPs in spleen, suggested possible macrophage-based internalization. Macrophage position and function in splenic domains confer them unique phenotypes thus corroborating tumor killing properties of MGF-AuNPs-pretreated macrophage cells, as observed in the co-culture experiments involving PC-3 cells (Fig. 9 and Scheme 3). These experimental findings are of significance because they lend experimental evidence on the unique characteristic of this nanomedicine agent to exploit trophic macrophages to subvert innate and adaptive immune responses capable of destroying malignant cells.

Targeting NF-κB signaling pathway, induction of polarization of macrophages to anti-tumor phenotype by inhibiting NF-κB phosphorylation, ability to promote the levels of anti-tumor cytokines, such as IL-12 and TNF-α, as discussed above—individually and collectively—infer the immunomodulatory features of MGF-AuNPs. In vivo therapeutic efficacy studies of MGF-AuNPs in tumor model was an obvious next step to evaluate whether the various immunomodulatory parameters, as observed in vitro, would be translated under the more complex in vivo tumor profiles in tumor bearing mice. We have therefore, undertaken detailed therapeutic efficacy of MGF-AuNPs in prostate tumor bearing SCID mice as discussed below.

### Therapeutic efficacy of MGF-AuNPs in treating prostate tumor

**Figure 11:**
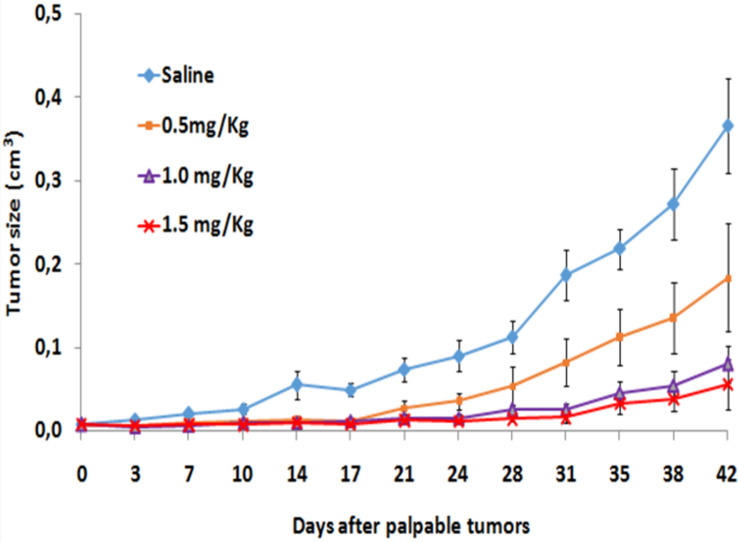
MGF-AuNPs inhibit PC-3 tumor growth in SCID mice. The SCID mice were divided in groups of seven mice each and implanted with PC3 cells subcutaneously in the right flanks. The tumors were allowed to grow till visible and palpable. Once palpable, the mice were randomized and treated with MGF-AuNPs (0.5 mg/kg, 1.0 mg/kg and 1.5 mg/kg body weight). These experiments were terminated once the untreated control mice (SCID) were morbid and started to lose weight. The tumor volumes were measured during the course of the study and plotted.

We have used severe combined immunodeficient (SCID) male mice bearing a flank model of human prostate cancer, derived from a subcutaneous implant of 10 million PC-3 cells, for the therapeutic efficacy and pharmacokinetic studies. In our evaluations, unilateral solid tumors were allowed to grow for three weeks, and animals were randomized (denoted Day 0) into control and treatment groups (n = 7) with no significant differences in tumor volume (0.0076 ± 0.08 to 0.0083 ± 0.04 cm^3^). In vivo dosing involved administering on day 0 three doses of MGF-AuNP (0.5 mg/kg bw, 1.0 mg/kg bw and 1.5 mg/kg bw—in 100 μL Dulbecco’s PBS) intraperitoneally, while the control SCID mice received only 100 μL Dulbecco’s PBS/saline. This treatment regimen was performed twice per week. Tumors were then measured twice each week until the end of the study (Day 42). Figure 11 shows results from the MGF-AuNPs-treated human prostate cancer bearing SCID mice. Within two weeks (Day 14), tumor growth in the treated animals started slowing down with respect to the control animals. Day 17, post administration of MGF-AuNPs (1.5 mg/kg bw), tumor volumes were two-fold lower (*p* < 0.005) for treated animals as compared to the control group. Three weeks, post administration of after MGF-AuNPs (1.5 mg/kg bw), tumor volumes for the control animals were fully six-fold greater with respect to those for the MGF-AuNPs-treated group (*p* < 0.0001; 0.37 ± 0.05 *vs*. 0.06 ± 0.02 cm^3^)—suggesting > 85% reduction in the overall tumor volume for the treated group. This significant therapeutic effect was maintained throughout the 42 days long study. Tumors harvested from the treatment group consisted largely of necrotic tissue, indicating extensive death of tumor cells.

Interestingly, biodistribution studies suggested that very low percentage of MGF-AuNPs reached the tumor tissue while most of the therapeutic agent was found in the spleen (Fig. 10). It is important to note that SCID mice lack T and B cells which are needed for the growth of human prostate tumors. In this context, the remarkable therapeutic efficacy, as shown in Fig. 11, therefore, suggests the ability of MGF-AuNPs to target the tumor microenvironment and, thus ensue modification of macrophages as the primary mode of therapeutic action of this nanomedicine agent.

The above data provides experimental validation to our hypothesis that MGF-AuNPs reeducate the macrophages to eliminate the tumors. It may be noted that the initial antitumor response is based on macrophages direct interaction with tumor cells and inflammatory cytokine release. These macrophages, after initial interaction with tumor cells, present antigens to T cells. Tumors in most cases modify the macrophages, which then help in progression of these tumors and suppress the secondary immune response. Therapeutic efficacy data, in conjunction with immunomodulatory characteristics as discussed in previous sections, demonstrate that our new therapeutic agent MGF-AuNPs can restore that balance where macrophages reeducation can lead to tumor growth inhibition. However, in our future investigations, we will be evaluating the effect of MGF-AuNPs on tumor inhibition using immune-sufficient mice.

The compelling therapeutic efficacy data, as summarized above, was further corroborated through evaluation of angiogenesis inhibitory effects of MGF-AuNPs in vivo. Angiogenesis is ubiquitous in tumor growth, invasion, progression, and metastasis of a vast majority of human cancers^120^. Therefore, targeting this process may potentially halt the growth and spread of cancers^121^. Some of the prominent FDA approved angiogenesis inhibitors currently used in cancer therapy (with their mode of action) include: Bevacizumab (VEGF-A antibody); Ramucirumab (VEGFR2 antibody); Sunitinib (Tyrosine Kinase Inhibitor); Sorafenib (Tyrosine Kinase Inhibitor); Pazopanib (Tyrosine Kinase Inhibitor); Lenvatinib (Tyrosine Kinase Inhibitor); and Cabozantinib (Tyrosine Kinase Inhibitor)^121^. Angiogenesis inhibitors act through direct interference in blood vessel growth. A well-established mode of action of angiogenesis inhibitors manifests binding to VEGF and/or its receptors^122^. Angiogenesis inhibitors are also known to bind to various cell surface receptors, VEGF receptors 1 and 2 which participate in angiogenesis, or they block blood vessel growth through strong and selective binding interactions with proteins in the downstream signaling pathways. Our rationale behind these studies stemmed from a variety of immunomodulatory features of MGF-AuNPs, as discussed in the preceding sections. There is considerable experimental evidence supporting that angiogenesis inhibitors are immunomodulatory and are capable of suppressing tumor growth^123^ immune system.

### Inhibition of angiogenesis in vivo

The compelling immunomodulatory effects, in controlling growth of tumors in vivo, prompted us to investigate the effects of MGF-AuNPs on angiogenesis in tumor tissues obtained through in vivo therapeutic efficacy experiments as discussed above. These studies were performed through immunostaining using the CD31 antibody because it has high specific affinity for vascular endothelial cells. Twelve fields (at 400x), from each prostate tumor xenografts, were analyzed to determine the average number of vessels per field (micro-vessel density = MVD). The findings from these studies indicated a significant reduction of MVD in samples collected from the MGF-AuNPs (1.5 mg/kg bw) treated animals as compared to the control group (saline treated) (Figure S7A–C). Manifestation of angiogenesis in MGF-AuNPs-treated tumor tissues, taken in concurrence with a plethora of immunomodulatory data discussed above, infers the immunomodulatory angiogenesis inhibitor characteristics of this nanomedicine agent exerting stimulatory effects on the immune system.

## Conclusions

Our studies lend credible experimental evidence demonstrating that inhibition of the receptor activator of NF-κB, by the new nanomedicine agent MGF-AuNPs, prevents prostate cancer development. The ability of MGF-AuNPs to target NF-κB signaling pathway will provide an attractive therapeutic strategy for the treatment of various forms of advanced cancers. As a vast majority of patients with solid tumors require therapeutic approaches with capabilities to reprogram the local immunosuppressive tumor milieu in order to revive antitumor immunity, detailed immunotherapeutic investigations, and results reported herein, provide compelling evidence on macrophage targeting abilities of the new MGF-AuNP nanoceutical. Our pre-clinical in vitro and in vivo studies have shown that MGF-AuNPs effectively target tumor-associated macrophages (TAMs) which abundantly infiltrate most solid tumors. TAMs-targeting strategies of MGF-AuNPs have been effectively used to initiate macrophage re-education from pro tumor M2 macrophages to antitumor M1 phenotype—thus eliminating cancer cells, restrict tumor growth and metastasis. Overall, our green nanotechnology discoveries, which have resulted in the development of a new generation of phytochemical-encapsulated nanomedicine agent (MGF-AuNP), provide further new insights on the therapeutic potential of TAM targeting nanoceuticals to improve immunotherapies.

## Methods

### Materials

Mangiferin, Sodium tetrachloroaurate (III) dihydrate, MTT (3-(4,5-dimethyl thiazol-2-yl)-2,5-diphenyl tetrazolium), dynasore reagent and Chlorpromazine (CPZ) were obtained from Sigma (St. Louis, MO, USA). RPMI, fetal calf serum, TryplE, Trypan blue, DAPI (4′,6-diamidino-2-phenylindole), 2,7-dichlorofluorescin diacetate (DCFH-DA), mouse IgG isotype control, and laminin receptor antibody (MLuC_5_) were obtained from ThermoFisher Scientific, USA. FITC Annexin V Apoptosis Detection Kit was obtained from BD Pharmingen, USA. X-22 anti-clathrin antibody (ab2731), anti-Caveolin-1 antibody (ab2910), anti-fibronectin antibody (ab18265), and in vitro angiogenesis assay kits (ab204726) were obtained from Abcam, USA. GFP-CERTIFIED Apoptosis/ Necrosis detection kit (ENZ-51002) was obtained from Enzo Life Sciences, Inc., USA. Phospho-NF-κB p65 (Ser536) (93H1) Rabbit mAb (Alexa Fluor488 Conjugate) Kit was obtained from Cell Signaling Technology, USA. Double distilled water was used throughout the experiment.

### Cell lines

The human prostate cancer (PC-3), human aortic endothelial cells (HAECs), and mouse macrophages (RAW 264.7) cell lines were obtained from the American Type Culture Collection (ATCC; Manassas, VA), and cultured by the University of Missouri Cell and Immunobiology Core facility using procedures recommended by ATCC. Recent gene therapy results, using in vitro and in vivo models, unequivocally suggest that PC-3 cells are excellent models for investigations related to drug targeting approaches for treating castration-resistant prostate cancer (CRPC)^124–131^.

### Synthesis of Mangiferin conjugated gold nanoparticles (MGF-AuNPs)

The Mangiferin gold nanoparticles (MGF-AuNPs) were produced by mixing of 4.2 mg mangiferin (MGF) in 6 mL of doubly deionized (DI) water. The solution was stirred at 100 °C for 10 min to dissolve the MGF into water to get a clear solution^132^. Sodium tetrachloroaurate (100 µL of 0.1 M) was added to the reaction mixture to produce gold nanoparticles (AuNPs). Change in color from yellow to burgundy wine red indicated the formation of MGF-AuNPs in the homogeneous reaction mixture. The MGF-AuNPs were characterized by various instrumentation techniques including, UV–Vis spectrophotometry^133^, Zetasizer Nano S90, TEM^134^ and ICP-MS^135^. For various in vitro and in vivo investigations, the treatment concentrations were calculated based on the amount of gold present in MGF-AuNPs. The amount of Au was calculated by ICP-MS technique^136^.

### Cellular internalization and trafficking pathway

The endocytosis mode of MGF-AuNPs was investigated by pre-blocking various receptors onto PC-3 cells. The optimum dose and incubation time were determined by incubating PC-3 cells with various concentrations of Mangiferin conjugated gold nanoparticles (MGF-AuNPs) at different time points. Further, the cell trafficking pathway of MGF-AuNPs was evaluated by using various receptor blocking agents to confirm the clathrin and or caveolae mediated endocytosis. Chlorpromazine (CPZ) and X-22 anti-clathrin antibodies were chosen to inhibit clathrin-mediated endocytosis. Anti-Caveolin-1 antibody was chosen to block/inhibit caveolae-mediated uptake. Internalization of MGF-Gold nanoparticles were monitored by two independent techniques: (i) Cytoviva dark field fluorescence microscopy and; (ii) transmission electron microscopy (TEM)^134^. For the dark field microscopic study, ultra clean and sterile cover slip was kept in 6 well plate to grow the PC-3 cells. For the study by TEM technique^134^, cells were grown on the plate without any coverslip.

Briefly, PC-3 cells (10^6^/mL) were seeded into 6 well plates in RPMI medium and incubated for 24 h in CO_2_ incubator at 37 °C. The cells were pre-incubated with the inhibitors as follows: PBS (control), chlorpromazine hydrochloride (10 µM; 20 min), X-22 anti-clathrin antibody (3 µg/mL; 60 min), anti-caveolin-1 antibody (3 µg/mL; 60 min), mouse IgG isotype control (10 µg/mL; 60 min), anti-fibronectin antibody (3 µg/mL; 60 min), and laminin receptor antibody (ABLR) (10 µg/mL; 60 min). The cells were incubated with all the inhibitors in CO_2_ incubator at 37 °C. Post incubation, cells were washed with 1XPBS twice followed by incubation with MGF-AuNPs (41 µM) for 60 min in the CO_2_ incubator at 37 °C. The samples were prepared by the following techniques:

### Dark field microscopic technique

After incubation, cells were washed 10 times with 1X PBS, and fixed with 4% paraformaldehyde (PFA) for 10 min in the CO_2_ incubator at 37 °C. Cells were further washed 2 times with 1X PBS and slides were prepared by using DAPI nuclear dye and observed under CytoViva dark field microscope coupled with dual mode fluorescence. Cell morphology was initially observed, followed by the uptake of nanoparticles. Images were captured via Dage Imaging Software.

### TEM technique

After incubation, cells were washed 10 times with 1X PBS, trypsinized and centrifuged into pellets, and fixed with 2% glutaraldehyde, 2% paraformaldehyde in sodium cacodylate buffer (0.1 M). The cells were further fixed with 1% buffered osmium tetroxide in 2-Mercaptoethanol buffer and dehydrated in graded acetone series and embedded in Epon-Spurr epoxy resin. Sections were cut at 85 nm using a diamond knife (Diatome, Hatfield PA). The sections were stained with Sato’s triple lead stain and 5% aqueous uranyl acetate for organelle visualization. The samples, as prepared above, were examined on JEOL 1400 TEM microscope (JEOL, Peabody, Mass.) operated at 80 kV at the University of Missouri’s Electron Microscopy Core Facility^134^.

MGF-AuNPs were explored for their selective affinity toward tumor cells by incubating with the same concentrations and time points as used for the normal endothelial cells (HAECs).

### Cell viability assay

The effect of MGF-AuNPs on prostate cancer (PC-3) and normal human aortic endothelial (HAECs) cells viability was determined using MTT assay (Sigma) over a period of 48 and 72 h. The intensity of developed color was measured by micro plate reader (Molecular device, USA) operating at 570 nm wavelength. Percent cell viability was calculated by using the formula: (T/C) × 100, where C = Absorbance of control, T = Absorbance of treatment. The IC-50 values were calculated using the Origin software^137^. Starch stabilized gold nanoparticles (S-AuNPs) as well as Gum-Arbaic stabilized gold nanoparticles (GA-AuNPs) were used as control NPs group for the in vitro experiments in all the cell viability assay to demonstrate minimal/no effect of control group on the cells.

### Apoptosis assay

PC-3 cells were incubated with MGF-AuNPs for 24 h and the experiment was performed according to the manufacture’s protocol (FITC Annexin V Apoptosis Detection Kit I). The samples were analyzed by FACScan flow cytometry (FACSort, Becton Dickinson, USA). For each sample, 30,000 ungated events were acquired^138^.

### Assessment of apoptotic and necrotic cell morphology

PC-3 cells were incubated with MGF-AuNPs for 24 h and the experiment was performed according to the manufacture’s protocol (GFP-CERTIFIED) Apoptosis/ Necrosis detection kit). Briefly, the PC-3 cells after treatment with either MGF-AuNPs or Staurosporin were incubated with the apoptosis and necrosis detection reagent for 10 min. The slides were prepared and visualized under fluorescent microscope with a dual filter set for Cyanine-3 (Ex/Em: 550/570 nm), 7-AAD (Ex/Em: 546/647) and GFP/FITC (Ex/Em: 488/514) (Olympus, USA).

### In vitro anti-angiogenesis assay

In vitro anti-angiogenesis effect of MGF-AuNPs on HAECs cells was determined using tube formation assay. The test was performed according the manufacture’s protocol (In vitro angiogenesis assay kit). Briefly, matrigel was coated in 96 well plate and the plates were incubated for 30 min at 37 °C. HAECs cells and test samples were added into the same plates and incubated for 24 h for tube formation analysis. The images were captured by fluoresce microscope, (Olympus, Center Valley, PA, USA) at 4 × magnification after 24 h^139^.

### NF-κB measurement

The Phospho-NF-κB p65 (Ser536) (93H1) Rabbit mAb (Alexa Fluor488 Conjugate (catalog number 3033) Kit was used to study the effect of MGF-AuNPs on the expression of NF-κB. Briefly, PC-3 cells were seeded into 6 well plate at a density of 10^6^ cells/mL and were incubated for 24 h. The cells were treated with MGF-AuNPs (40 µM) for 18 h and post-treated with TNF-α (0.1 nM) for another 30 min at 37 °C. The assay was performed as per kit instructions and the results were analyzed by FACScan flow cytometry (FACSort, Becton Dickinson, USA) with a minimum of 10,000 events being recorded.

### Macrophage MGF-AuNPs uptake studies

RAW 264.7 macrophages were cultured in DMEM + 10% FBS in a 75 cm^2^ flask. The cells at a density of 10^6^ were plated in 6 well plates overnight for adherence. The cells were then replenished with fresh medium and incubated with MGF-AuNPs (40 µM) for 60 min. Cells were then analyzed for the presence of gold nanoparticles by CytoViva dark field microscopy.

### The effect of MGF-AuNPs on NF-κB inhibition in RAW 264.7 macrophages

Cells were preincubated with MGF-AuNPs for 2 h followed by treatment with either LPS (100 ng/mL-positive control) or RANKL (10 ng/mL) for 30 min. Cells were lysed and lysates were run on polyacrylamide gel electrophoresis and transferred onto nitrocellulose membrane. These membranes were then probed with phospho-NF-κB antibody and NF-κB from Cell Signaling Technologies^140^.

### Flow cytometry for NF-κB in RAW macrophages

RAW 264.7 cells (a murine monocyte/macrophage cell line, ATCC) were plated in 6-well culture plates in DMEM culture media (Gibco) supplemented with 10% fetal calf serum (Sigma-Aldrich) and 1% penicillin–streptomycin and stored overnight at 37 °C and 5% CO_2_. The confluent macrophages were then pre-treated with different doses of MGF-AuNPs (0, 8, 16, and 32 µg/mL) and S-AuNPs (32 µg/mL) for 3 h. Subsequently the cells were washed with DMEM (1%FBS 1% P/S) to remove uninternalized nanoparticles and then treated with LPS (100 ng/mL) or TNF-α (20 ng/mL) for 45 min in DMEM (1% FBS 1% P/S). After incubation with LPS or TNF-α, the cells were suspended by scraping and transferred to 5 mL round-bottom polystyrene tubes (Falcon). The collected samples were centrifuged at 1500 rpm for 5 min, the supernatant was removed, and the cells were fixed with 4% paraformaldehyde in PBS for 15 min at room temperature. The cells were then washed in PBS, centrifuged at 1500 rpm for 5 min, and the supernatant was decanted. The cells were permeabilized for 30 min at room temperature in 90% methanol. After permeabilization, the cells were washed and incubated in 100μL of anti-NF-κB p65 (Ser536) antibody (Cell Signaling, 5733) at 1:50 dilution for 1 h at room temperature in the dark. Samples were centrifuged at 1500 rpm for 5 min, washed with 2 mL of 1% FBS PBS. Flow cytometry data was acquired using the BD LSRFortessa X-20 Flow Cytometer and analyzed using Flowjo software.

### Cytokine analysis by real time PCR following treatment of RAW 264.7 cells with MGF-AuNPs

RAW 264.7 cells were cultured in 6 well plates overnight for adherence followed by the treatment with MGF-AuNPs for 4 h. LPS and Starch stabilized AuNPs (S-AuNPs) were used as positive and negative control respectively. The cells were lysed, and RNA was isolated using RNA isolation kit from Qiagen (Germantown, MD). RNA was then analyzed for IL-12, TNF-α, IL-10 and IL-6 using real time PCR^141^.

### Effect of MGF-AuNPs treated macrophages on prostate cancer cell proliferation

PC-3 cells at a density of 10^5^ cells/well were plated in 6 well plate overnight for adherence. Subsequently, RAW 264.7 macrophages were treated with MGF-AuNPs (4 µM) for 18–24 h. The macrophages were washed to remove unbound MGF-AuNPs in order to avoid the direct effect of MGF-AuNPs on cancer cells. In the meantime, the PC-3 cells were labelled with carboxyfluorescien succinimidyl ester (CFSE) to assess their proliferation. The macrophages were then co-cultured with PC-3 cells using a ratio of 10:1 (1 part of PC-3 cells to 10 part of macrophages) for 72 h. The co-culture was observed under the fluorescent microscope and pictures taken. We observed significant reduction in the proliferation of PC-3 cells when cocultured with macrophages which were pre-treated with MGF-AuNPs^142^.

### Animal studies

All in vivo work has been performed at an IACUC approved laboratory and in accordance with ARRIVE guidelines for animal welfare.

### Ethics declarations

All experiments of MGF-AuNPs involving animals were approved by the Institutional Animal Care and Use Committees of the Harry S. Truman Memorial Veterans Hospital and the University of Missouri and were performed in accordance with the Guide for the Care and Use of Laboratory Animals under an IACUC approved protocol number 8767. Severe combined immuno-deficient SCID (ICR-SCID) male mice show a severe combined immunodeficiency (from Taconic Farms, Hudson, New York) were used for the therapeutic study. The mice used in our investigations weighed 24–27 g.

### Description of animal procurement, housing, and grouping

Animals were maintained on a 12 h light–dark cycle and had access to sterilized standard chow and water *ad libidum*. Animals were allowed to acclimate for 7–10 days prior to initiation of work. Human prostate cancer cell line PC-3 was obtained from the American Type Culture Collection (ATCC; Manassas, VA), and cultured according to ATCC recommendations by the University of Missouri Cell and Immunobiology Core facility. Small Cell Neuroendocrine Carcinoma (SCNC) is a hormone resistant aggressive cancer which does not respond to classic androgen therapy. PC-3 is a SCNC cell line which is highly metastatic and does not express classic hormone receptors and hence are resistant to hormone therapy. Moreover, the patients treated with hormone therapy tend to relapse of SCNC^143–145^. Mice received ear tag identifiers under inhalational anesthesia (isoflurane/oxygen) followed by unilateral, subcutaneous hind flank inoculations of 10 × 10^6^ PC-3 cells suspended in 0.1 mL of sterile Dulbecco’s phosphate buffered saline (DPBS) and Matrigel (2:1, *v*:*v*). Solid tumors were allowed to develop over a period of 3 weeks, and animals were randomized (Day 0) into control and treatment groups (n = 7) having no significant difference in tumor volumes (*p* = 0.64; Student’s t-test) or body weights (*p* = 0.17). Tumor volumes were estimated from caliper measurements using the formula V = length × width × depth. On Day 8, animals in the treatment group received intraperitoneal administrations of MGF-AuNP agent in DPBS (100 μL) while under inhalational anesthesia in doses as outlined in the following section. Similarly, control animals received 30 μL of saline intraperitoneally. No significant difference (*p* = 0.93) in tumor volume or body weight (*p* = 0.21) was noted between the groups. Tumor volumes, body weights and health status were then determined twice each week. At the end of the study (Day 42), mice were euthanized by cervical dislocation, and blood sample was collected by cardiac puncture. Samples of spleen, liver, tumor, and blood were harvested, weighed and submitted to the University of Missouri Neutron Activation Analysis (NAA) facility at the University of Missouri Research reactor (MURR) for the accurate quantification of gold in various tissues by NAA analysis.

NOTE: Although male mice have been selected in our investigations, it is important to note that this is a xenograft model, thus murine gender is not anticipated to significantly influence tumor biology. Importantly, as we are studying prostate cancer, the use of male mice only is appropriate.

### In vivo bio-distribution study by neutron activation analysis (NAA)

To assess the gold content in various tissue in SCID mice (n = 7). 1.5 mg/ kg bw of MGF-AuNPs were administered in these mice for seven weeks, while control mice (n = 7) did not receive any treatment with MGF-AuNPs. Tumor tissue, spleen, liver and blood were harvested upon euthanization, put into chloridometer sample vials and dried for approximately 48 h at 100–120 °C. Dried tissue mass of approximately 0.5–1.0 g was placed into polyethylene vials (used for control of counting geometry). We estimated the amount of gold in various tissue samples as described previously through neutron activation analysis (NAA) techniques^38^.

### In vivo therapeutic efficacy study

Antitumor efficacy of MGF-AuNPs was evaluated by using prostate tumor xenografts in SCID mice as developed above. Briefly, SCID male mice were subcutaneously inoculated with 10 × 10^6^ PC-3 cells (suspended in 0.1 mL of sterile DPBS and Matrigel (2∶1, v:v)) in the right hind flank under inhalation anesthesia (isoflurane/oxygen). After inoculation, tumors were allowed to grow for 2–3 weeks, at which time the tumors were measured by digital caliper measurements and calculated as length × width × height. The mice were randomly divided into four groups (n = 7/group) with no significant difference in tumor volume, randomization was generated using the standard = RAND() function in Microsoft Excel, and the day of randomization was considered the day zero of therapy study (Table 1). On day zero, mice were given intraperitoneal injections as follows: Group 1: saline treated (100 μL); Group 2: MGF-AuNPs treated (0.5 mg/kg bw); Group 3: MGF-AuNPs treated (1.0 mg/kg bw) and Group 4: MGF-AuNPs treated (1.5 mg/kg bw)—all in 100 μL Dulbecco’s PBS. Using this regimen, animals were treated twice per week until the end of the study (42 days). The animals were monitored for their tumor volume, body weight and health effects until they were sacrificed at the end of the study. The fifth group (n = 7) was kept as control group (no tumor and no treatment) and served as a control for complete blood count (CBC) values and body weight measurements. Animals were sacrificed at the end of study. Measurement of tumor volumes were carried out twice each week until the end of the study (Day 42).

**Table 1:** ARRIVE description.

Items	Recommendation	Our response with section/line number or reason for not reporting
Study design	1. For each experiment provide brief details of study design including: (a) The groups being compared, including control groups. If no control group has been used, the rationale should be stated (b) The experimental unit (e.g. a single animal, litter, or cage of animals)	1. Full details of in vivo Therapeutic efficacy Studies Design (a and b): All in vivo work has been performed at an IACUC approved laboratory and in accordance with ARRIVE guidelines for animal welfare. Animal studies were approved by the Institutional Animal Care and Use Committees of the Harry S. Truman Memorial Veterans Hospital and the University of Missouri and were performed in accordance with the Guide for the Care and Use of Laboratory Animals under an IACUC approved protocol number 8767 We have over three decades of experience in conducting hypothesis driven cancer research with in vivo models using tumor bearing SCID mice to minimize discomfort and adverse effects in study animals (both control and treated animals). Here are a few representative publications where we have outlined similar in vivo investigations which have been accepted by the global scientific peers: (1) Ravi Shukla, Nripen Chanda, and Kattesh V. Katti et al.: ^198^AuNP-EGCg for prostate cancer therapy: Proceedings of the National Academy of Sciences Jul 2012, 109 (31) 12426–12431; https://doi.org/10.1073/pnas.1121174109 (2) Nripen Chanda, Vijaya Kattumuri, Kattesh V. Katti, et al.: Bombesin functionalized gold nanoparticles show in vitro and in vivo cancer receptor specificity: Proceedings of the National Academy of Sciences May 2010, 107 (19) 8760– 8765; https://doi.org/10.1073/pnas.1002143107 (3) Nripen Chanda, Para Kan, Kattesh V. Katti, et al.; Radioactive gold nanoparticles in cancer therapy: therapeutic efficacy studies of GA-198AuNP nanoconstruct in prostate tumor–bearing mice: Nanomedicine: Nanotechnology, Biology and Medicine, Volume 6, Issue 2, 2010, Pages 201–209, ISSN 1549-9634, https://doi.org/10.1016/j.nano.2009.11.001 Brief description of in vivo investigations: Male SCID mice (4–5 weeks of age; Taconic Farms, Hudson, NY) were housed in a temperature and humidity-controlled pathogen-free barrier facility NOTE: Although male mice have been selected in our investigations, it is important to note that this is a xenograft model, thus murine gender is not anticipated to significantly influence tumor biology. Importantly, as we are studying prostate cancer, the use of male mice only is appropriate Description of animal procurement, housing, and grouping: Animals were maintained on a 12 h light–dark cycle and had access to sterilized standard chow and water *ad libidum*. Animals were allowed to acclimate for 7–10 days prior to initiation of work. Human prostate cancer cell line PC-3 was obtained from the American Type Culture Collection (ATCC; Manassas, VA), and cultured according to ATCC recommendations by the University of Missouri Cell and Immunobiology Core facility. Mice received ear tag identifiers under inhalational anesthesia (isoflurane/oxygen) followed by unilateral, subcutaneous hind flank inoculations of 10 × 10^6^ PC-3 cells suspended in 0.1 mL of sterile Dulbecco’s phosphate buffered saline (DPBS) and Matrigel® (2:1, *v*:*v*). Solid tumors were allowed to develop over a period of 3 weeks, and animals were randomized (Day 0) into control and treatment groups (n = 7) having no significant difference in tumor volumes (*p* = 0.64; Student’s t-test) or body weights (*p* = 0.17). Tumor volumes were estimated from caliper measurements using the formula V = length × width × depth. On Day 8, animals in the treatment group received intraperitoneal administrations of MGF-AuNP agent in DPBS (100 µL) while under inhalational anesthesia in doses as outlined in the following section. Similarly, control animals received 100 µL of saline intraperitoneally. No significant difference (*p* = 0.93) in tumor volume or body weight (*p* = 0.21) was noted between the groups. Tumor volumes, body weights and health status were then determined twice each week. At the end of the study (Day 42), mice were euthanized by cervical dislocation, and blood sample was collected by cardiac puncture. Samples of spleen, liver, tumor, and blood were harvested, weighed and submitted to the University of Missouri Neutron Activation Analysis (NAA) facility at the University of Missouri Research reactor (MURR) for the accurate quantification of gold in various tissues by NAA analysis
Sample size	2 (a) Specify the exact number of experimental units allocated to each group, and the total number in each experiment. Also indicate the total number of animals used (b) Explain how the sample size was decided. Provide details of any a priori sample size calculation, if done	Details of Sample Size with descriptions for a and b: As outlined above, SCID mice were randomly divided into four groups (n = 7/group) with no significant difference in tumor volumes On day zero, mice were administered intraperitoneal injection of MGF-AuNP agent in DPBS (100 µL) (or saline for the control group) as follows: Group 1-saline treated; Group 2-MGF-AuNPs treated (0.5 mg/kg bw); Group 3-MGF-AuNPs treated (1.0 mg/kg bw); Group 4-MGF-AuNPs treated (1.5 mg/kg bw) The fifth group (n = 7) was kept as control group (no tumor and no treatment) and served as a control for the evaluation of complete blood count (CBC) values and body weight measurements. Animals were subjected to vaporizer 5% isoflurane and sacrificed at the end of study (Day 42) The above-mentioned sample size is based on consultations with our biostatistician who confirmed that a n = 7 in different treatment and control groups, as elaborated above, would provide scientifically credible statistical significance to our preclinical data
Inclusion and exclusion criteria	3 (a) Describe any criteria used for including and excluding animals (or experimental units) during the experiment, and data points during the analysis. Specify if these criteria were established a priori. If no criteria were set, state this explicitly (b) For each experimental group, report any animals, experimental units or data points not included in the analysis and explain why. If there were no exclusions, state so (b) For each analysis, report the exact value of n in each experimental group	Inclusion criteria: On the choice of the animal model, we have used Male SCID mice in our investigations NOTE: Although male mice have been selected in our investigations, it is important to note that this is a xenograft model, thus murine gender is not anticipated to significantly influence tumor biology. Importantly, as we are studying prostate cancer, the use of male mice only is appropriate Experimental and control groups: SCID mice were randomly divided into four groups (n = 7/group) with no significant difference in tumor volumes On day zero, mice were administered intraperitoneal injection of MGF-AuNP agent in DPBS (100 µL) (or saline for the control group) as follows: Group 1-saline treated Group 2-MGF-AuNPs treated (0.5 mg/kg bw) Group 3-MGF-AuNPs treated (1.0 mg/kg bw) Group 4-MGF-AuNPs treated (1.5 mg/kg bw) The fifth group (n = 7) was kept as control group (no tumor and no treatment) and served as a control for the evaluation of complete blood count (CBC) values and body weight measurements. Animals were subjected to vaporizer 5% isoflurane and sacrificed at the end of study (Day 42) There were no exclusions in our investigations. Exclusion criteria: N/A
Randomization	4 (a) State whether randomization was used to allocate experimental units to control and treatment groups. If done, provide the method used to generate the randomization sequence (b) Describe the strategy used to minimize potential confounders such as the order of treatments and measurements, or animal/cage location. If confounders were not controlled, state this explicitly	Mice received ear tag identifiers under inhalational anesthesia (isoflurane/oxygen) followed by unilateral, subcutaneous hind flank inoculations of 10 × 10^6^ PC-3 cells suspended in 0.1 mL of sterile Dulbecco’s phosphate buffered saline (DPBS) and Matrigel® (2:1, *v*:*v*). Solid tumors were allowed to develop over a period of 3 weeks, and animals were randomized (Day 0) into control and treatment groups (n = 7) having no significant difference in tumor volumes (*p* = 0.64; Student’s t-test) or body weights (*p* = 0.17). Tumor volumes were estimated from caliper measurements using the formula V = length × width × depth. On Day 8, animals in the treatment group received intraperitoneal administrations of MGF-AuNP agent in DPBS (100 µL) while under inhalational anesthesia in doses as outlined in the following section. Similarly, control animals received 100 µL of saline intraperitoneally. No significant difference (*p* = 0.93) in tumor volume or body weight (*p* = 0.21) was noted between the groups. Tumor volumes, body weights and health status were then determined twice each week All animal facilities at the Harry S. Truman Memorial Veterans Hospital and the University of Missouri, Columbia, Missouri, were visited daily by the veterinarian care staff, inspected by the institutional animal care and use committee members throughout the investigation Mice were examined daily, and removed from the study if unresponsive to supportive care, moribund, if weight loss is > 20% body weight or if tumor size > 5 cm^3^ with poor body condition (hunched posture and loss of > 20% body weight, or easily palpated exoskeleton) or with lassitude with written protocols to euthanatize such animals to minimize animal discomfort
Blinding	5. Describe who was aware of the group allocation at the different stages of the experiment (during the allocation, the conduct of the experiment, the outcome assessment, and the data analysis)	All experiments of MGF-AuNPs involving animals were approved by the Institutional Animal Care and Use Committees (IACUC, protocol number 8767) of the Harry S. Truman Memorial Veterans Hospital and the University of Missouri were performed according to the Guide for the Care and Use of Laboratory Animals. All animal facilities at the Harry S. Truman Memorial Veterans Hospital and the University of Missouri, Columbia, Missouri, were visited daily by the veterinarian care staff, inspected by the institutional animal care and use committee members throughout the investigation The animal modeling staffs and veterinarians, who conducted our animal experiments, have over 20 years of experience in all aspects of pharmaceutical testing through placebo-controlled, blinded pre clinal investigations in tumor bearing mice. Such data has formed the basis for seeking approval for Phase 1 trials of various drugs discovered by us in the past. In the current investigation, as reported in our manuscript, our staff have exercised due care and caution to perform blinded experiments in prostate tumor bearing xenografts in SCID mice with our nanomedicine agents MGF-AuNP with saline in controls
Outcome measures	6 (a) Clearly define all outcome measures assessed (e.g. cell death, molecular markers, or behavioral changes) (b) For hypothesis-testing studies, specify the primary outcome measure, i.e. the outcome measure that was used to determine the sample size	Outcome measures: Morbidity: As accepted by the institutional Animal Care and Use Committees (IACUC, protocol number 8767) of the Harry S. Truman Memorial Veterans Hospital and the University of Missouri Guide for the Care and Use of Laboratory Animals, we defined morbidity to include any animal where one of the following conditions exists: the tumor volume exceeds 5 cm^3^, ulceration of the overlying skin of the tumor is observed, ulceration of the tumor itself is observed, body weight-loss of more than 20% is noted, and/or significant illness/depression (whether or not related to the experimental protocol) is observed. Animals exhibiting any signs of morbidity, as defined, will be sacrificed immediately to minimize and alleviate any unnecessary pain and suffering. Mortality was evaluated by measuring any differences in the total survival times between groups as a function of study termination time These studies required 7 mice per experimental group to provide meaningful statistical results based on the expectation of accurately detecting a 20% difference in experimental tumor groups Hypothesis validation and expected primary outcome: The overall hypothesis was to validate the antitumor characteristics of the experimental nanomedicine agent. The following groups of control and tumor bearing mice were administered intraperitoneal injections of MGF-AuNP agent in DPBS (100 µL) (or saline for the control group) as follows: Group 1-saline treated Group 2-MGF-AuNPs treated (0.5 mg/kg bw) Group 3-MGF-AuNPs treated (1.0 mg/kg bw) Group 4-MGF-AuNPs treated (1.5 mg/kg bw) The fifth group (n = 7) was kept as control group (no tumor and no treatment) and served as a control for the evaluation of complete blood count (CBC) values and body weight measurements. Animals were subjected to vaporizer 5% isoflurane and sacrificed at the end of study (Day 42) 50–80% reduction in tumor volumes in the treated groups, as compared to the control group, with minimal/no adverse toxic side effects, (Groups 2–4) was the expected outcome of this hypothesis driven investigation Throughout the study, the animals were monitored for their tumor volume (groups 1–4), body weight and overall health (group 1–5). Mice were examined daily, and removed from the study if unresponsive to supportive care, moribund, if weight loss is > 20% body weight or if tumor size > 5 cm^3^ with poor body condition (hunched posture and loss of > 20% body weight, or easily palpated exoskeleton) or with lassitude with written protocols to euthanatize such animals to minimize animal discomfort At the end of the study (day 42) animals were subjected to vaporizer 5% isoflurane and before being sacrificed and following samples were collected from group 1–4, blood, tissues (spleen, liver, and tumor) Blood samples from all groups were used for complete blood count (CBC) values The tissues (spleen, liver, tumor and blood) were submitted to the University of Missouri Neutron Activation Analysis (NAA) facility at the University of Missouri Research reactor (MURR) for the accurate quantification of gold in various tissues by NAA analysis
Statistical methods	7 a. Provide details of the statistical methods used for each analysis, including software used. b. Describe any methods used to assess whether the data met the assumptions of the statistical approach, and what was done if the assumptions were not met	Mortality was evaluated by measuring any differences in the total survival times between groups as a function of study termination time. These studies required 7 mice per experimental group to provide meaningful statistical results based on the expectation of accurately detecting a 20% difference in experimental tumor groups All experimental data are described as mean ± SEM. Statistical analysis was carried out using the one-way analysis of variances (ANOVA) using Graph Pad Prism software. *P* < 0.05 was considered significant
Experimental animals	8 (a) Provide species-appropriate details of the animals used, including species, strain and substrain, sex, age or developmental stage, and, if relevant, weight (b) Provide further relevant information on the provenance of animals, health/immune status, genetic modification status, genotype, and any previous procedures	Species-appropriate details: (a) Male SCID mice (4–5 weeks of age; Taconic Farms, Hudson, NY) were housed in a temperature and humidity-controlled pathogen-free barrier facility NOTE: Although male mice have been selected in our investigations, it is important to note that this is a xenograft model, thus murine gender is not anticipated to significantly influence tumor biology. Importantly, as we are studying prostate cancer, the use of male mice only is appropriate Male SCID mice (4–5 weeks of age; Taconic Farms, Hudson, NY) weighing 20–25 g in weight were housed in a temperature and humidity-controlled pathogen-free barrier facility (b) Description of animal procurement, housing, and grouping: Animals were maintained on a 12 h light–dark cycle and had access to sterilized standard chow and water *ad libidum*. Animals were allowed to acclimate for 7–10 days prior to initiation of work. Human prostate cancer cell line PC-3 was obtained from the American Type Culture Collection (ATCC; Manassas, VA), and cultured according to ATCC recommendations by the University of Missouri Cell and Immunobiology Core facility. Mice received ear tag identifiers under inhalational anesthesia (isoflurane/oxygen) followed by unilateral, subcutaneous hind flank inoculations of 10 × 10^6^ PC-3 cells suspended in 0.1 mL of sterile Dulbecco’s phosphate buffered saline (DPBS) and Matrigel® (2:1, *v*:*v*). Solid tumors were allowed to develop over a period of 3 weeks, and animals were randomized (Day 0) into control and treatment groups (n = 7) having no significant difference in tumor volumes (*p* = 0.64; Student’s t-test) or body weights (*p* = 0.17). Tumor volumes were estimated from caliper measurements using the formula V = length × width × depth. On Day 8, animals in the treatment group received intraperitoneal administrations of MGF-AuNP agent in DPBS (100 µL) while under inhalational anesthesia in doses as outlined in the following section. Similarly, control animals received 100 µL of saline intraperitoneally. No significant difference (*p* = 0.93) in tumor volume or body weight (*p* = 0.21) was noted between the groups. Tumor volumes, body weights and health status were then determined twice each week. At the end of the study (Day 42), mice were euthanized by cervical dislocation, and blood sample was collected by cardiac puncture. Samples of spleen, liver, tumor, and blood were harvested, weighed and submitted to the University of Missouri Neutron Activation Analysis (NAA) facility at the University of Missouri Research reactor (MURR) for the accurate quantification of gold in various tissues by NAA analysis
Experimental animals	9 For each experimental group, including controls, describe the procedures in enough detail to allow others to replicate them, including: (a) What was done, how it was done and what was used (b) When and how often (c) Where (including detail of any acclimatization periods) (d) Why (provide rationale for procedures)	Full experimental details of all nnimal studies reported in our manuscript: Ethics Committee Approvals: All in vivo work has been performed at an IACUC approved laboratory and in accordance with ARRIVE guidelines for animal welfare. Animal studies were approved by the Institutional Animal Care and Use Committees of the Harry S. Truman Memorial Veterans Hospital and the University of Missouri and were performed in accordance with the Guide for the Care and Use of Laboratory Animals under an IACUC approved protocol number 8767 NOTE: We have over three decades of experience in conducting hypothesis driven cancer research with in vivo models using tumor bearing SCID mice to minimize discomfort and adverse effects in study animals (both control and treated animals). Here are a few representative publications where we have outlined similar in vivo investigations which have been accepted by the global scientific peers: (4) Ravi Shukla, Nripen Chanda, and Kattesh V. Katti et al.: ^198^AuNP-EGCg for prostate cancer therapy: Proceedings of the National Academy of Sciences Jul 2012, 109 (31) 12426–12431; https://doi.org/10.1073/pnas.1121174109 (5) Nripen Chanda, Vijaya Kattumuri, Kattesh V. Katti, et al.: Bombesin functionalized gold nanoparticles show in vitro and in vivo cancer receptor specificity: Proceedings of the National Academy of Sciences May 2010, 107 (19) 8760– 8765; https://doi.org/10.1073/pnas.1002143107 (6) Nripen Chanda, Para Kan, Kattesh V. Katti, et al.; Radioactive gold nanoparticles in cancer therapy: therapeutic efficacy studies of GA-198AuNP nanoconstruct in prostate tumor–bearing mice: Nanomedicine: Nanotechnology, Biology and Medicine, Volume 6, Issue 2, 2010, Pages 201–209, ISSN 1549-9634; https://doi.org/10.1016/j.nano.2009.11.001 Justification for the Use of Animals: There are no in vitro tests that can be used to substitute for the complex tumor microenvironment occurring in vivo when testing experimental candidates for their effectiveness as prostate cancer therapy agents. Our investigations of therapeutic effectiveness studies, of a new nanomedicine agent, MGF-AuNP, in vivo using human prostate tumor xenografts in SCID mice are necessary. Therefore, the SCID mice model with prostate tumor xenografts, as described in our manuscript, represents the most widely accepted and the best model for pre-clinical evaluations of novel therapeutic strategies ultimately intended for use in treating human prostate tumor patients All experiments of MGF-AuNPs involving animals were approved by the Institutional Animal Care and Use Committees (IACUC, protocol number 8767) of the Harry S. Truman Memorial Veterans Hospital and the University of Missouri were performed according to the Guide for the Care and Use of Laboratory Animals We have used severely compromised immunodeficient (SCID) mice bearing a flank model of human prostate cancer derived from a subcutaneous implant of 10 million PC-3 cells for therapeutic efficacy and pharmacokinetic studies (from Taconic Farms, Hudson, New York) were used for the therapeutic study. The mice used in our investigations weighed 24–27 g In vivo bio-distribution study by Neutron Activation Analysis (NAA). To assess the gold content in tissue in SCID mice (n = 7). 1.5 mg/kg bw MGF-AuNPs were administered in these mice for seven weeks, while control mice (n = 7) did not receive any treatment with MGF-AuNPs. Tumor, spleen, liver and blood were harvested upon euthanization (vaporizer 5% isoflurane), put into chloridometer vials and dried for approximately 48 h at 100–120 °C. Dried tissue mass of approximately 0.5–1.0 g was placed into polyethylene vials (used for control of counting geometry). We estimated the amount of gold in various tissue samples as described previously In vivo therapeutic efficacy study. Antitumor efficacy of MGF-AuNPs was evaluated by developing prostate tumor model (in SCID male mice). The SCID male mice were subcutaneously inoculated with 10 × 10^6^ PC-3 cells (suspended in 0.1 mL of sterile DPBS and Matrigel® (2∶1, v:v)) in the right hind flank under inhalation anesthesia (isoflurane/ oxygen). After inoculation, tumors were allowed to grow for 2–3 weeks, at which time the tumors were measured by digital caliper measurements and calculated as length × width × height. The mice were randomly divided into four groups (n = 7/group) with no significant difference in tumor volume, randomization was generated using the standard = RAND() function in Microsoft Excel, and the day of randomization was considered the day zero of therapy study. On day zero, mice were given intraperitoneal injections as follows: Group 1: saline treated (100 µL); Group 2: MGF-AuNPs treated (0.5 mg/kg bw); Group 3: MGF-AuNPs treated (1.0 mg/kg bw) and Group 4: MGF-AuNPs treated (1.5 mg/kg bw)—all in 100 µL Dulbecco’s PBS. Using this regimen, animals were treated twice per week until the end of the study (42 days). The animals were monitored for their tumor volume, body weight and health effects until they were sacrificed at the end of the study. The fifth group (n = 7) was kept as control group (no tumor and no treatment) and served as a control for complete blood count (CBC) values and body weight measurements. Animals were sacrificed at the end of study. Measurement of tumor volumes were carried out twice each week until the end of the study (Day 42). Within two weeks (Day 14), tumor growth in the treated group (with MGF-AuNPs at 1.5 mg/kg bw), appeared to be slowing with respect to the controls. After 17 days of post administration (dose of MGF-AuNPs at 1.5 mg/kg bw), tumor volumes were two- fold lower (*p* < 0.005) for treated animals compared to controls. This significant therapeutic effect was maintained throughout our observational study. Tumor volumes for the control animals were fully six-seven-fold greater with respect to those for the MGF-AuNPs-treated group (*p* < 0.0001; 0.37 ± 0.05 *vs*. 0.06 ± 0.02 cm^3^) groups—at three weeks, post administration of after MGF-AuNPs (1.5 mg/kg bw). These observations were indicative of > 85% reduction in the overall tumor volume for the treated group. This profound therapeutic efficacy was observed throughout the 42 days long study. Tumors harvested from the treatment group consisted largely of necrotic tissue, indicating extensive death of tumor cells The tissues (spleen, liver, tumor tissue and blood) were isolated from prostate tumor xenografts and were submitted to the University of Missouri Neutron Activation Analysis (NAA) facility at the University of Missouri Research reactor (MURR) for the accurate quantification of gold in various tissues by NAA analysis
Results	10 For each experiment conducted, including independent replications, report: (a) Summary/descriptive statistics for each experimental group, with a measure of variability where applicable (e.g. mean and SD, or median and range). (b) If applicable, the effect size with a confidence interval	Full description of Results/Summary (taken directly from our manuscript): In our evaluations, unilateral solid tumors were allowed to grow for three weeks, and animals were randomized (denoted Day 0) into control and treatment groups (n = 7) with no significant differences in tumor volume. In vivo dosing involved administering on day 0 three doses of MGF-AuNP (0.5 mg/kg bw, 1.0 mg/kg bw and 1.5 mg/kg bw—in 100 µL Dulbecco’s PBS) intraperitoneally, while the control SCID mice received only 100 µL Dulbecco’s PBS/saline. Tumors were then measured twice each week until the end of the study (Day 42). Figure 11 shows results from the MGF-AuNPs-treated human prostate cancer bearing SCID mice. Within two weeks (Day 14), tumor growth in the treated animals started slowing down with respect to the control animals. Day 17, post administration of MGF-AuNPs (1.5 mg/kg bw), tumor volumes were two-fold lower (*p* < 0.005) for treated animals as compared to the control group. Three weeks, post administration of after MGF-AuNPs (1.5 mg/kg bw), tumor volumes for the control animals were fully six-fold greater with respect to those for the MGF-AuNPs-treated group (*p* < 0.0001; 0.37 ± 0.05 *vs*. 0.06 ± 0.02 cm^3^)—suggesting > 85% reduction in the overall tumor volume for the treated group. This significant therapeutic effect was maintained throughout the 42 days long study. Tumors harvested from the treatment group consisted largely of necrotic tissue, indicating extensive death of tumor cells

The tissues (spleen, liver, tumor tissue and blood) were isolated from prostate tumor xenografts and were submitted to the University of Missouri Neutron Activation Analysis (NAA) facility at the University of Missouri Research reactor (MURR) for the accurate quantification of gold in various tissues by NAA analysis.

### Statistical analysis

All experimental data are described as mean ± SEM. Statistical analysis was carried out using the one-way analysis of variances (ANOVA) using Graph Pad Prism software. *P* < 0.05 was considered significant.

## Supplementary Information

Supplementary Information.

Supplementary Figure 1.

Supplementary Figure 2.

Supplementary Figure 3.

Supplementary Figure 4.

Supplementary Figure 5.

Supplementary Figure 6.

Supplementary Figure 7.

## Abbreviations

ABLR: Laminin receptor antibody
ARRIVE: Animal Research: Reporting of In Vivo Experiments
BSA: Bovine serum albumin
BMDM: Bone-marrow-derived macrophage
Cad11: Cadherin-11
Cav-1: Caveolin-1
CBC: Complete blood count
CPZ: Chlorpromazine
CRPC: Castration-resistant prostate cancer
CFSE: Carboxyfluorescien succinimidyl ester
DAPI: 4′,6-Diamidino-2-phenylindole
DCFH-DA: 2,7-Dichlorofluorescin diacetate
DPBS: Dulbecco’s phosphate buffered saline
DLS: Dynamic Light Scattering
DU-145: Androgen receptor positive human prostate cancer cells
EDS: Energy-dispersive X-ray spectroscopy
EGCG: Epigallocatechin gallate
EGFR: Epidermal growth factor
EMT: Epithelial-mesenchymal transition
FACS: Fluorescence-Activated Cell Sorting
FGFR: Fibroblast growth factor
Fn-3: Anti-fibronectin AB
FITC: Fluorescein isothiocyanate
HSA: Human serum albumin
HAECs: Human aortic endothelial cells
IAEA: United Nation’s International Atomic Energy Agency
ICP-MS: Inductively Coupled Plasma Mass Spectrometry
IKK: IκB kinase
IκBs: NF-κB proteins
LNCaP: Lymph node carcinoma of the prostate cell line
M1: Anti-tumor macrophages
M2: Pro-tumor macrophages
MDSCs: Myeloid-derived suppressor cells
MGF: Mangiferin
MGF-AuNPs: Mangiferin functionalized gold nanoparticles
MMP: Matrix metalloproteinase
MLuC5: Laminin receptor antibody
MPS: Mononuclear phagocyte system
MVD: Micro-vessel density
MURR: University of Missouri Research reactor
NAA: Neutron Activation Analysis
NF-kB: Nuclear factor kappa-light-chain-enhancer of activated B cells
NK: Natural killer
LPS: Lipopolysaccharide
PBS: Phosphate buffered saline
PC-3: Human prostate tumor cells
PCR: Polymerase chain reaction
PDGFR: Platelet derived growth factor
PFA: Paraformaldehyde
PI: Propidium iodide
PXRD: Powder X ray diffraction
RAW 264.7: Murine macrophage cells
RPMI: Roswell Park Memorial Institute
S-AuNPs: Starch-stabilized gold nanoparticles
SCID: Severely combine immune-deficient
SCNC: Small Cell Neuroendocrine Carcinoma
SPR: Surface plasmon resonance
TAMs: Tumor associated macrophages
TEM: Transmission Electron Microscopy
TNF-α: Tumor necrosis factor alpha
VEGFR: Vascular endothelial growth factor
X-22: Anti-clathrin AB
