# Supplementary material for: Green nanotechnology of MGF-AuNPs for immunomodulatory intervention in prostate cancer therapy
Source: Sci Rep. 2021 Aug 18;11:16797. doi: 10.1038/s41598-021-96224-8 (PMC8373987; doi:10.1038/s41598-021-96224-8)
Supplement: Supplementary file 1 — Supplementary Information. [file 41598_2021_96224_MOESM1_ESM.doc]

**Supplementary Materials Section**

**NOTE: All new additions/revisions are coded with red colored text**

**Green Nanotechnology of MGF-AuNPs for**

**Immunomodulatory Intervention in Prostate Cancer Therapy**

Menka Khoobchandani^1,2^, Aslam Khan^3^, Kavita K Katti^1^, Velaphi C. Thipe^4^, Amal. Y. Al-Yasiri^5,6^, Darsha K. D. MohanDoss^7^, Michael B. Nicholl^8^, Ademar B. Lugão^4^, Chetan P. Hans^9^, Kattesh V. Katti^1,10,11^*

^1^Department of Radiology, **Institute of Green Nanotechnology,** University of Missouri, Columbia, MO, 65212, USA

^2^Department of Radiation Oncology, Washington University School of Medicine, 4511 Forest Park Ave, St. Louis, MO 63108

^3^Department of Biochemistry, University of Missouri, Columbia, MO, 65212, USA

^4^Laboratório de Ecotoxicologia - Centro de Química e Meio Ambiente - Instituto de Pesquisas Energéticas e Nucleares (IPEN) - Comissão Nacional de Energia Nuclear- IPEN/CNEN-SP, Butantã, São Paulo - SP, Brasil

^5^Nuclear Science and Engineering Institute (NSEI), University of Missouri, Columbia, MO 65211, USA

^6^University of Baghdad, College of Dentistry, Baghdad, Iraq

^7^Dhanvantari Nano Ayushadi Pvt Ltd, No. 8/34, Neelakanta Mehta Street, T. Nagar, Chennai – 600017-India

^8^South Texas VA Health Care San Antonio, Texas, USA

^9^Department of Medicine-Cardiology, University of Missouri, Columbia, MO, 65212, USA

^10^**Department of Physics, ^11^University of Missouri Research Reactor (MURR),** University of Missouri, Columbia, MO, 65212, USA

*Correspondence: E mail: KattiK@health.missouri.edu

**Methods**

**Characterization of nanoparticles by TEM.** Transmission electron microscopic (TEM) images were obtained on a JEOL 1400 TEM (JEOL, LTE, Tokyo, Japan) available at the University of Missouri Electron Microscopy Core facility. The absorption measurements were attained by UV-Vis spectrophotometer (Varian Cary 50 conc, USA). The hydrodynamic size and zeta potential were obtained using a Zetasizer Nano S90 (Malvern Instruments Ltd. USA). The concentration of gold metal in nanoparticles were estimated by inductively coupled plasma mass spectrometry (ICP-MS), Perkin Elmer, MA, USA. The presence of gold metal in MGF-AuNPs was characterized using energy-dispersive X-ray spectroscopy (EDS).^1–3^

**Characterization of nanoparticles by X-ray diffraction technique.** Liquid nanoparticle dispersions in water were spread out on disposable glass slides and allowed to evaporate overnight under ambient conditions. The resulting residues were scraped off the slide with a steel razor blade and pressed between two sheets of weighing paper by hand to form sub-millimeter sized pellets. The prepared samples were mounted on polyimide single crystal sample loops. X-ray diffraction data were measured on a Bruker X8 Prospector (Bruker AXS, Madison, WI, USA) single crystal diffractometer using Cu-Kα radiation from a microfocus source with multilayer optics (λ = 1.54060 Å, power = 45 kV, 0.60 mA). A series of 15 4-min-long photographs were taken while rotating the sample 360° about the phi axis, giving a total irradiation time of one hour. X-ray intensities were recorded across the 2θ of 28.5° to 60.5° using an Apex II CCD area detector situated at a crystal-to-detector distance of 10.0 cm and with the detector remaining stationary throughout the measurement. Photographic data were reduced by radially integrating a 33°-wide sector of the photograph in shells of 0.02° in 2θ using the Bruker Apex3 software suite.^1^

***In vitro* stability analysis.** The stability of MGF-AuNPs were confirmed by mixing gold nanoparticles with various biological solutions such as aqueous solutions of 1% NaCl, 0.5% cysteine, 0.2 M histidine, 0.5% human serum albumin (HSA), 0.5% bovine serum albumin (BSA), and pH7. The stability of the conjugates was measured by monitoring the surface plasmon resonance (SPR)^4^ at different time points for a week. A negligible/no change in SPR frequency confirmed stability and the retention of nanoparticulate composition in all mixtures. The MGF-AuNPs was also tested for their stability at different dilutions in the DI water using UV-visible spectrophotometry.^2^

**Results and Discussion**

**Characterization of MGF-AuNPs.**

The MGF-AuNPs were characterized by a combination of techniques including UV-Visible Spectrophotometry, Dynamic Light Scattering (DLS), Transmission Electron Microscopy (TEM) and Inductively Coupled Plasma Mass Spectrometry (ICP-MS). The UV-visible spectrophotometric analysis indicated a surface plasmon resonance (SPR) absorption at ~535 nm (Figure S1A), thus inferring the successful synthesis of AuNPs. The core size of MGF-AuNPs, obtained by TEM, indicated that the nanoparticles are spherical and nearly mono-disperse with metallic core size of 35±2 nm (Figure S1). The results obtained by DLS measurements revealed that MGF-AuNPs exhibited a hydrodynamic size of 55±5 nm and a zeta potential -40±2 mV (Table S1). Hydrodynamic size of 55±5 nm, which is greater than the core metallic size (35±2 nm) confirmed efficient encapsulation of Mangiferin around the gold nanoparticles. Preventing nanoparticles from aggregation is vitally important in the design and development of gold nanoparticles for biomedical applications. Kinetics of aggregation are dictated by electronic charge or through sterically demanding encapsulants. A significantly higher negative zeta potential (ζ) value of -40±2 mV, as observed for MGF-AuNPs, clearly suggests that these nanoparticles possess optimum electrostatic repulsion to remain stable in solution for extended periods. Inductively coupled plasma mass spectrometry (ICP-MS) indicated that MGF-AuNPs contained 327 ppm of gold. Full Physicochemical parameters of MGF-AuNPs are summarized in Table S1.

**Table S1: Physicochemical data parameters of MGF-AuNPs**

| **Sample** | **Absorbance (nm)** | **Core size by TEM (nm)** | **Hydrodynamic size by DLS (nm)** | **Zeta potential (mV)** | **[Au] in AuNPs**  **(by ICP-MS, ppm)** |
| --- | --- | --- | --- | --- | --- |
| MGF-AuNPs | 535 nm | 35±2 | 55±5 | -40±2 | 327 |

Note. TEM: Transmission electron microscopy, ICP-MS: inductively coupled plasma mass spectrometry, DLS: dynamic light scattering, and MGF: Mangiferin.

Energy-dispersive X-ray spectroscopy (EDS) analysis of MGF-AuNPs provided information regarding the presence of gold (Figure S2). Additional characterization of MGF-AuNPs was performed by recording the Powder X ray diffraction (PXRD) pattern (Figure S3). As shown in Figure S3, PXRD shows four peaks across the region of 28.5° to 60.5°. The lowest angle peak at 29.5° is a detector artifact which can be easily spotted from the X-ray diffraction photographs. The two major peaks at 38.32° and 44.46° match the known PXRD peaks for Au metal in this region. The NaCl (2 0 0) peak is visible 31.86°, while the NaCl (2 2 0) peak can be observed as a shoulder on the Au (2 0 0) peak with a maximum roughly at 45.60°.

***In vitro* stability study of MGF-AuNPs.** The *in vitro* stability of MGF-AuNPs was evaluated by incubating solutions of nanoparticles with various biological media including 0.5% cysteine, 0.2M histidine, 0.5% human serum albumin (HSA), 0.5% bovine serum albumin (BSA), 1% NaCl and pH 7 buffer solutions. Changes in SPR was used as a measure of stability in various media at different time points.^5–8^ As shown in Figure S4A, MGF-AuNPs exhibited no measurable changes in the position/shape of SPR, in various media throughout the measurement period. These observations suggest that the Mangiferin encapsulation creates a network of strongly hydrogen bonded corona around gold nanoparticles, thus affording excellent *in vitro* stability for over a week within various biological media (Figure S4A). *In vitro* stability of AuNPs, at dilutions that mimic *in vivo* biological conditions, is important for their applications in molecular imaging and therapy.^7,9–13^ Therefore, we have tested the stability of MGF-AuNPs at various dilutions. As depicted in Figure S4B, MGF-AuNPs exhibited robust stability at extreme dilutions which are typical of cellular concentrations encountered under *in vivo* conditions.

**Effects of S-AuNPs and GA-AuNPs on prostate tumor and normal HAECs cell viability.** Starch stabilized gold nanoparticles as well as Gum-Arabic stabilized gold nanoparticles were used as control NPs group for the *in vitro* experiments in all the cell viability assays to demonstrate minimal/no effect of control group on the cells over a period of 72 hr (Figure S5).

**Toxicity profile of MGF-AuNPs *in vivo*.** The body weight and blood parameter of all the experimental animals (SCID mice) were measured to evaluate the toxicity of all the treatments groups. In this study, normal control group (5^th^) was added which were not treated and not inoculated with tumor cells. The body weight in the group of MGF-AuNPs (1.5 mg/kg bw) treatment were found similar to the normal control group (Figure S7A). However, the body weight in the saline treated control group was found lower as compared to the normal control group.

Blood parameters were compared between the treatment and control groups with baseline levels obtained from a fifth group of SCID mice that received no manipulations. Analysis of variance followed by a post hoc Dunnett’s test was employed. Red blood cell and white blood cells counts between the three groups varied only slightly, by approximately 10%, and the hematocrit was not different between the three groups (Figure S7B). Platelet levels were elevated by 8% from baseline for the untreated tumor bearing animals (686 ± 29 x 10^3^ / µL vs. 815 ± 100 x 10^3^ / µL; p < 0.05), while those from the MGF-AuNPs group (743 ± 84 x 10^3^ / µL) showed no significant difference (p > 0.05).

**Figure legends:**

**Figure S1.** Characterization of MGF-AuNPs by (A) UV-Visible spectra, (B) HR-TEM images, (C) Core size distribution histogram employing image J software (D) TEM images. Note: TEM: transmission electron microscopy. HR-TEM: high resolution TEM.

**Figure S2:** Energy Dispersive Spectroscopy (EDS) analysis of MGF-AuNPs. Image (A) showing the presence of Au element, and image (B) mapping of Au element.

**Figure S3:** Powder X ray Diffraction (PXRD) pattern of evaporated residue for MGF-AuNPs.

**Figure S4.** (A) UV-Vis spectra showing *in vitro* stability of MGF-AuNPs in aqueous solutions after 1-week of incubation. (B) Spectra illustrate that the absorbance intensity is linearly proportional to MGF-AuNPs concentrations.

**Figure S5.** Green Nanotechnology architecture (A) Starch functionalized Gold Nanoparticles (S-AuNPs) and (B) Gum arabic functionalized Gold Nanoparticles (GA-AuNPs). MTT cell viability assay for prostate tumor cells and normal cells (C and D, respectively), S-AuNPs and GA-AuNPs no effect on both prostate tumor and normal cells.

**Figure S6:** MGF-AuNPs inhibit TNF-α induced NF-κB (p65) activation in PC-3 cells. PC-3 cells were cultured in 6 well plates for overnight followed by preincubation with MGF-AuNPs for 1 hr. The cells were washed and treated with TNF-α for 30 min. After treatment cells were harvested and permeabilized and stained with PE conjugated anti-NF-κB antibody for 45 min – 1 hr. The cells were washed and analyzed by flow cytometry.

**Figure S7:** Effect of MGF-AuNPs on animals’ health in terms of (A) body weight, (B) blood count and (C) MGF-AuNPs showing anti-angiogenesis effects. The body weight and blood data were compared with normal control group (no treatment and no tumor). Body weights were observed twice per week for all groups, for approximately 2-4 weeks. Animals were sacrificed at the end of the study. Tumor tissues were harvested and fixed with formalin. The tissues were utilized for immunohistochemistry analysis by staining with CD31 antibody to observe blood vessels.

**References**

1. Techane, S. D., Gamble, L. J. & Castner, D. G. X-ray photoelectron spectroscopy characterization of gold nanoparticles functionalized with amine-terminated alkanethiols. *Biointerphases* **6**, 98–104 (2011).

2. Gajendiran, M., Jainuddin Yousuf, S. M., Elangovan, V. & Balasubramanian, S. Gold nanoparticle conjugated PLGA–PEG–SA–PEG–PLGA multiblock copolymer nanoparticles: synthesis, characterization, in vivo release of rifampicin. *J. Mater. Chem. B* **2**, 418–427 (2014).

3. Brun, F., Travan, A., Accardo, A. & Paoletti, S. Characterization of silver nanoparticles for biomedical applications by means of quantitative analysis of tem micrographs - biomed 2010. *Biomed. Sci. Instrum.* **46**, 105–110 (2010).

4. Couture, M., Zhao, S. S. & Masson, J.-F. Modern surface plasmon resonance for bioanalytics and biophysics. *Phys. Chem. Chem. Phys.* **15**, 11190–11216 (2013).

5. Khoobchandani, M. *et al.* New approaches in breast cancer therapy through green nanotechnology and nano-ayurvedic medicine – pre-clinical and pilot human clinical investigations. *Int. J. Nanomedicine* **15**, 181–197 (2020).

6. Al-Yasiri, A. Y. *et al.* Mangiferin functionalized radioactive gold nanoparticles (MGF-198AuNPs) in prostate tumor therapy: green nanotechnology for production, in vivo tumor retention and evaluation of therapeutic efficacy. *Dalt. Trans.* **46**, 14561–14571 (2017).

7. Nune, S. K. *et al.* Green nanotechnology from tea: Phytochemicals in tea as building blocks for production of biocompatible gold nanoparticles. *J. Mater. Chem.* **19**, 2912–2920 (2009).

8. Katti, K. V. K. *et al.* Green Nanotechnology from Cumin Phytochemicals: Generation of Biocompatible Gold Nanoparticles. *Int. J. Green Nanotechnol. Biomed.* **1**, B39–B52 (2009).

9. Shukla, R. *et al.* Laminin receptor specific therapeutic gold nanoparticles ((AuNP)-Au-198-EGCg) show efficacy in treating prostate cancer. *Proc. Natl. Acad. Sci.* **109**, 12426 LP – 12431 (2012).

10. Chanda, N. *et al.* Bombesin functionalized gold nanoparticles show in vitro and in vivo cancer receptor specificity. *Proc. Natl. Acad. Sci.* **107**, 8760–8765 (2010).

11. Khoobchandani, M. *et al.* *Targeted Phytochemical-Conjugated Gold Nanoparticles in Cancer Treatment*. (Springer International Publishing, 2019). doi:10.1007/978-3-319-92399-4_3.

12. Chanda, N. *et al.* Radioactive gold nanoparticles in cancer therapy: therapeutic efficacy studies of GA-198AuNP nanoconstruct in prostate tumor–bearing mice. *Nanomedicine Nanotechnology, Biol. Med.* **6**, 201–209 (2010).

13. Fent, G. M. *et al.* Biodistribution of maltose and gum arabic hybrid gold nanoparticles after intravenous injection in juvenile swine. *Nanomedicine Nanotechnology, Biol. Med.* **5**, 128–135 (2009).
