# Supplementary figures and images for: Green nanotechnology of MGF-AuNPs for immunomodulatory intervention in prostate cancer therapy
Source: Sci Rep. 2021 Aug 18;11:16797. doi: 10.1038/s41598-021-96224-8 (PMC8373987; doi:10.1038/s41598-021-96224-8)

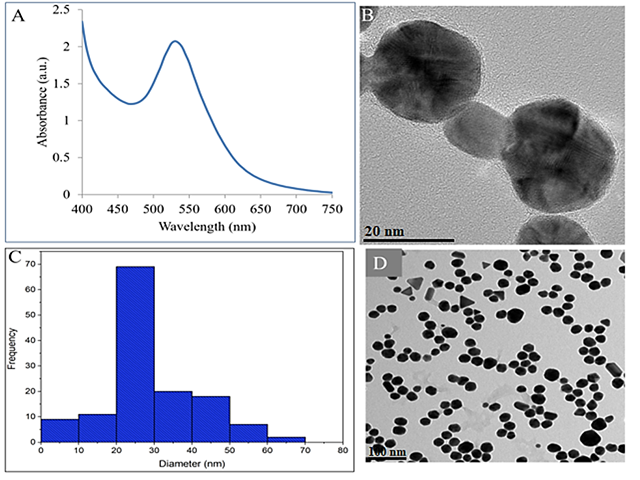

Supplement: Supplementary file 2 — Supplementary Figure 1. [file 41598_2021_96224_MOESM2_ESM.tif]

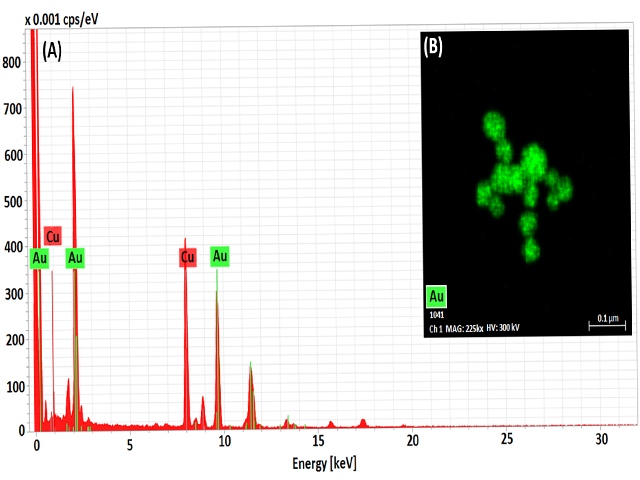

Supplement: Supplementary file 3 — Supplementary Figure 2. [file 41598_2021_96224_MOESM3_ESM.tif]

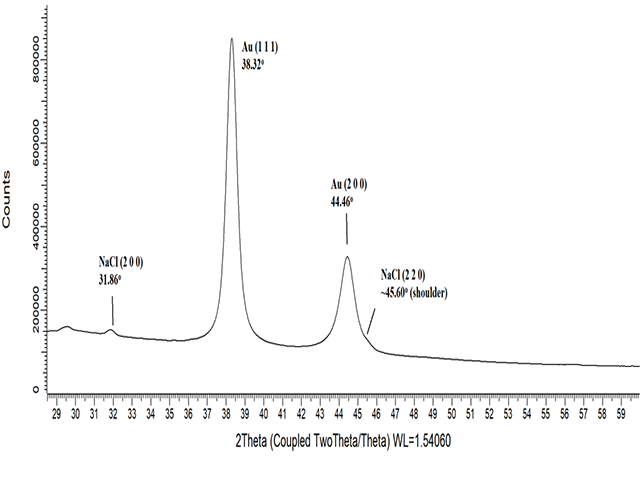

Supplement: Supplementary file 4 — Supplementary Figure 3. [file 41598_2021_96224_MOESM4_ESM.tif]

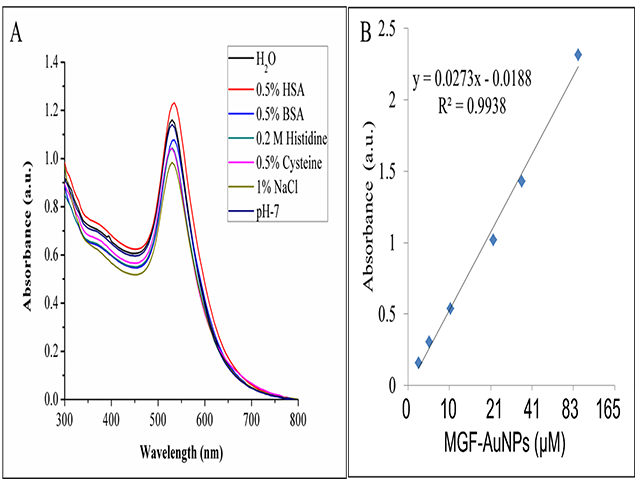

Supplement: Supplementary file 5 — Supplementary Figure 4. [file 41598_2021_96224_MOESM5_ESM.tif]

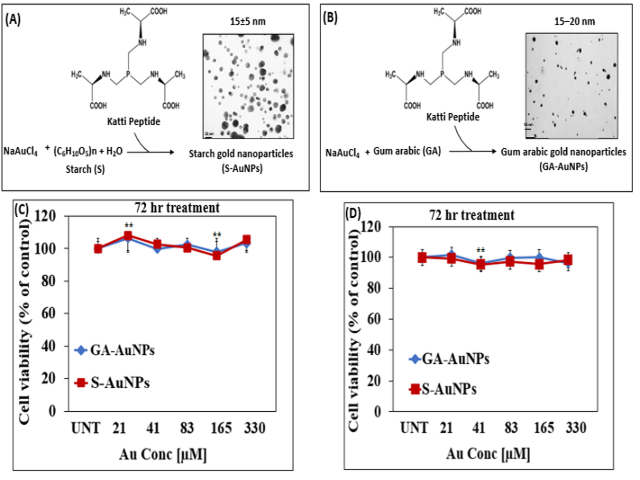

Supplement: Supplementary file 6 — Supplementary Figure 5. [file 41598_2021_96224_MOESM6_ESM.tiff]

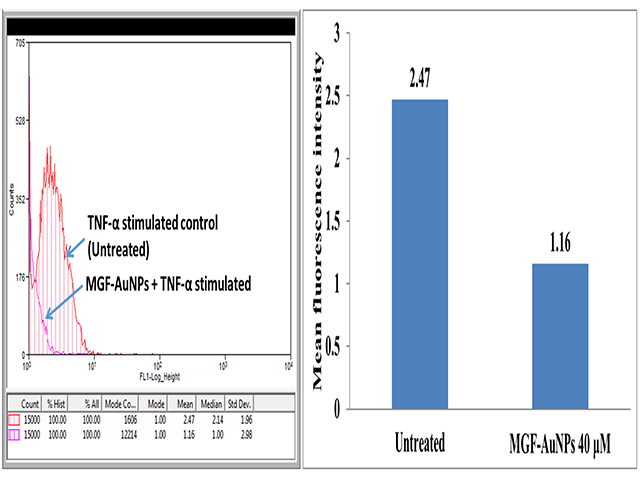

Supplement: Supplementary file 7 — Supplementary Figure 6. [file 41598_2021_96224_MOESM7_ESM.tif]

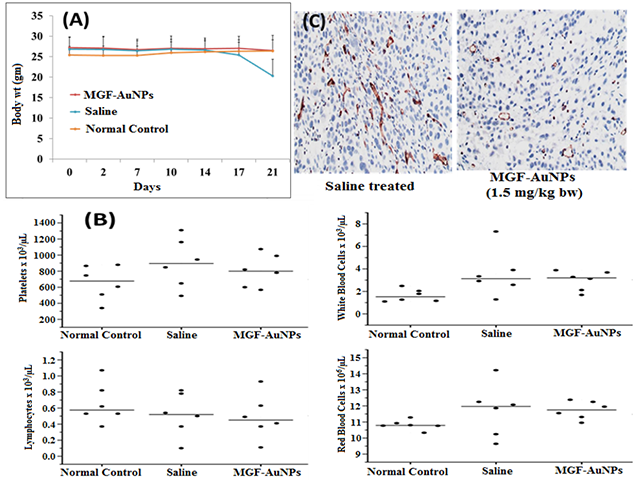

Supplement: Supplementary file 8 — Supplementary Figure 7. [file 41598_2021_96224_MOESM8_ESM.tif]
